# Catalog of MicroRNA Seed Polymorphisms in Vertebrates

**DOI:** 10.1371/journal.pone.0030737

**Published:** 2012-01-27

**Authors:** Minja Zorc, Dasa Jevsinek Skok, Irena Godnic, George Adrian Calin, Simon Horvat, Zhihua Jiang, Peter Dovc, Tanja Kunej

**Affiliations:** 1 Department of Animal Science, Biotechnical Faculty, University of Ljubljana, Domzale, Slovenia; 2 Centre for Mathematical and Computational Biology, Rothamsted Research, Harpenden, United Kingdom; 3 Department of Experimental Therapeutics and The Center for RNA Interference and Non-Coding RNAs, The University of Texas, M.D. Anderson Cancer Center, Houston, Texas, United States of America; 4 National Institute of Chemistry, Ljubljana, Slovenia; 5 Department of Animal Sciences, Washington State University, Pullman, Washington, United States of America; University of Barcelona, Spain

## Abstract

MicroRNAs (miRNAs) are a class of non-coding RNA that plays an important role in posttranscriptional regulation of mRNA. Evidence has shown that miRNA gene variability might interfere with its function resulting in phenotypic variation and disease susceptibility. A major role in miRNA target recognition is ascribed to complementarity with the miRNA seed region that can be affected by polymorphisms. In the present study, we developed an online tool for the detection of miRNA polymorphisms (miRNA SNiPer) in vertebrates (http://www.integratomics-time.com/miRNA-SNiPer) and generated a catalog of miRNA seed region polymorphisms (miR-seed-SNPs) consisting of 149 SNPs in six species. Although a majority of detected polymorphisms were due to point mutations, two consecutive nucleotide substitutions (double nucleotide polymorphisms, DNPs) were also identified in nine miRNAs. We determined that miR-SNPs are frequently located within the quantitative trait loci (QTL), chromosome fragile sites, and cancer susceptibility loci, indicating their potential role in the genetic control of various complex traits. To test this further, we performed an association analysis between the *mmu-miR-717* seed SNP rs30372501, which is polymorphic in a large number of standard inbred strains, and all phenotypic traits in these strains deposited in the Mouse Phenome Database. Analysis showed a significant association between the *mmu-miR-717* seed SNP and a diverse array of traits including behavior, blood-clinical chemistry, body weight size and growth, and immune system suggesting that seed SNPs can indeed have major pleiotropic effects. The bioinformatics analyses, data and tools developed in the present study can serve researchers as a starting point in testing more targeted hypotheses and designing experiments using optimal species or strains for further mechanistic studies.

## Introduction

MicroRNAs (miRNA) are non-coding RNA molecules with approximately 21 nucleotides in length, which play an important role in posttranscriptional regulation of mRNA. By binding to the target gene's complementary sequence of the 3′ untranslated region (3′UTR) they repress translation [1]. Changes in miRNA expression profiles have been identified in diseases, including several cancer types (reviewed in [2], [3]). Additionally, single nucleotide polymorphisms (SNPs) of miRNA precursors, their target sites, and silencing machinery were reported to interfere with miRNA function and they are likely to affect phenotypic variation, including disease susceptibility [4]. For example, genetic variants affecting the miRNA pathways were involved in diseases such as cancer, neurological disorders, muscular hypertrophy, gastric mucosal atrophy, cardiovascular disease, and type 2 diabetes [5]–[7]. The term miR-SNP refers to the variation that occurs in the miRNA gene sequence, while the miR-TS-SNP to the SNP that occurs in the miRNA target site (TS) or binding site [8]. Because one miRNA can have multiple targets, miR-SNPs would be expected to exhibit more profound and broader biological effects than miR-TS-SNPs [8]. SNPs in miRNA genes may alter their sequences and therefore enhance, diminish or even generate or cancel out their ability to bind to target sites [9]. Therefore, miR-SNPs could have an impact on the catalogue of miRNA targets, not only by disrupting the interaction of the mutant miRNA with its target genes, but also by creating illegitimate targets that are not targeted by the wild type miRNA [10].

The key binding location for translational suppression resides in the mature miRNA sequence, more accurately nucleotides 2–7 or 2–8 from the 5′ end of the miRNA, also called the seed region [8]. The minimal pairing requirement is a 6-nt match of the seed region (2–7 nucleotides), which can be extended to a 7-nt match (2–8 nucleotides) due to a highly conserved nucleotide position immediately upstream [11]. The causal effect of the miR-SNPs in the seed region (miR-seed-SNPs) on phenotypic variation has been shown recently; two groups discovered that a miR-seed-SNP in miR-96 was responsible for hearing loss in human and mouse [10], [12]. A genomic overlap of four layers was also identified consisting of growth associated quantitative trait locus (QTL), body mass associated *Gpc3* gene, miRNA gene (*mmu-miR-717*), and miR-seed-SNP identified in the lean mouse strain 129/Sv [13].

Polymorphisms within miRNA genes have been reported to be rare, with only approximately 10% (65/474 reported miRNAs at that time) of human pre-miRNAs having documented SNPs, and <1% (3/474) of miRNAs having SNPs in the functional seed region [9]. Similarly, a survey on 173 human miRNA genes revealed 10 SNPs in the pre-miRNAs but none in the seed region [14]. As such, the information about miR-seed-SNPs has received much less attention, while the data remains limited mostly to human and mouse, fragmented, and dispersed among various databases and publications: Patrocles [15], PolymiRTS [16], miRvar [17], and miRNASNP [18]. In contrast, miR-TS-SNPs that influence disease susceptibility, especially cancer, have been the subject of intense research in the last few years [19], [20]. Additionally, catalog of SNPs residing in miRNA binding regions has already been compiled [21]. Therefore, the aim of the present study was to develop the tool for detection of miRNA polymorphisms in vertebrates and assemble the information associated with SNPs residing within the miRNA seed region into a single catalog. This web-based public resource should enable faster and more targeted studies on miR-seed-SNP biology precluding a need for preliminary bioinformatics, mouse model and phenotype screens.

## Materials and Methods

### Development of the online tool for the detection of genetic variations within miRNA genes

A web based tool named “miRNA SNiPer” was developed for the detection of polymorphisms residing within miRNA genes in vertebrates. It accepts a list of miRNA genes and returns a table of variations within different regions of miRNA genes: pre-miRNA, mature, and seed region. The mature sequences are designated as “miR” and the precursor hairpins as “mir” [22]. The tool retrieves data from multiple sources: 1) miRNA gene sequences, genomic coordinates, and nomenclature from miRBase, release 18 (http://www.mirbase.org/) [23], 2) locations of miRNA seed regions from TargetScan, release 5.2 (http://www.targetscan.org/) [11], and 3) locations of genetic polymorphisms from Ensembl Variation database, release 64 (http://www.ensembl.org/) [24]. The assemblies from miRBase, TargetScan, and Ensembl Variation database were downloaded and locally inserted into a MySQL database. The tool is implemented as a CGI (Common Gateway Interface) script written in Perl. Script triggers SQL commands to the MySQL database to perform the searches of variations within miRNA genes. The tool miRNA SNiPer is available at http://integratomics-time.com/miRNA-SNiPer/.

### Catalog of the miR-seed polymorphisms

The developed tool miRNA SNiPer was used to generate an assembly of miR-seed polymorphisms in vertebrates (http://www.integratomics-time.com/miR-seed-SNPs/catalog/). The assembled list was supplemented with information relevant for further analysis from the literature (PubMed: http://www.ncbi.nlm.nih.gov/pubmed; Web of Science: http://apps.webofknowledge.com/)and from other sources. Validation status and allele frequencies were retrieved from the National Center for Biotechnology Information (NCBI) (http://www.ncbi.nlm.nih.gov/). Overlaps with host genes were retrieved from miRBase, release 18 [23]. Validated miRNA targets were extracted from TarBase v5.0 (http://diana.cslab.ece.ntua.gr/tarbase/) [25] and miRecords (http://mirecords.biolead.org) databases [26]. The list of assembled miR-seed polymorphisms was additionally verified with other online databases and tools.

### Physical characterization of the miRNA polymorphisms

Genomic distribution of miR-seed polymorphisms was presented on a genomic view (http://www.integratomics-time.com/miR-seed-SNPs/genomic_view/) using Flash GViewer web tool (http://gmod.org/wiki/Flashgviewer/). Overlap analysis of miRNAs comprising seed-SNPs with genomic fragile sites was performed using data retrieved from Ensembl *via* BioMart. Human and mouse QTL were retrieved from the Rat Genome Database (RGD) (http://rgd.mcw.edu/) [27] and chicken QTL were retrieved from Animal QTL Database, release 15 (http://www.animalgenome.org/cgi-bin/QTLdb/index/) [28].

### Functional characterization of the miR-seed polymorphisms

TargetScan Custom feature (http://www.targetscan.org/) was used to analyze whether the miR-seed-SNP cause the formation of seed regions annotated to different miRNA [11]. The information regarding the association between miRNAs with polymorphic seed regions and diseases was retrieved from miR2Disease (http://watson.compbio.iupui.edu:8080/miR2Disease/) [29] and published literature (PubMed).

### Seed SNP genotype to phenotype association analysis

Association between the mouse seed SNP in *mmu-miR-717* (rs30372501) and phenotypes was analyzed. Data for the genotype-phenotype association analysis was downloaded from the Mouse Phenome Database (MPD; http://phenome.jax.org/) [30]. A test was carried out on all phenotypic data from MPD consisting of 2586 traits in 35 groups (appearance and coat color, behavior, blood-clinical chemistry, blood-hematology, blood-lipids, blood-miscellaneous, body composition, body weight size and growth, bone, brain, breathing pattern, cancer, cardiovascular, cell and tissue damage, development, ear, endocrine, eye, gallbladder, immune system, ingestive preference, kidney, liver, local experiment parameter, longevity, metabolism, metastatic progression, mouse procurement, muscle, nervous system, neurosensory, reproduction, respiratory, sensory gating, and spleen). Seed SNP in *mmu-miR-717* (rs30372501 genotypic and phenotypic data was available for 14 inbred mouse strains (129S1/SvImJ, A/J, AKR/J, BALB/cByJ, C3H/HeJ, C57BL/6J, DBA/2J, FVB/NJ, KK/HlJ, MOLF/EiJ, NOD/ShiLtJ, NZW/LacJ, PWK/PhJ, and WSB/EiJ) consisting of a various number of measurements, ranging from one to 311 for each strain.

Statistical package SAS/STAT [31] was used for statistical analyses. The following linear model was used in the analysis:
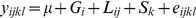
(1)where y_ijkl_ represents the observation for the traits, μ trait average, G_i_ fixed effect of genotype *mmu-miR-717* seed SNP rs30372501 (*i* = CC, TT), L_ij_ nested effect of strain (*j* = 1−14), S_k_ fixed effect of sex (*k* = f, m), and e_ijkl_ random error.

## Results and Discussion

**Figure 1:**
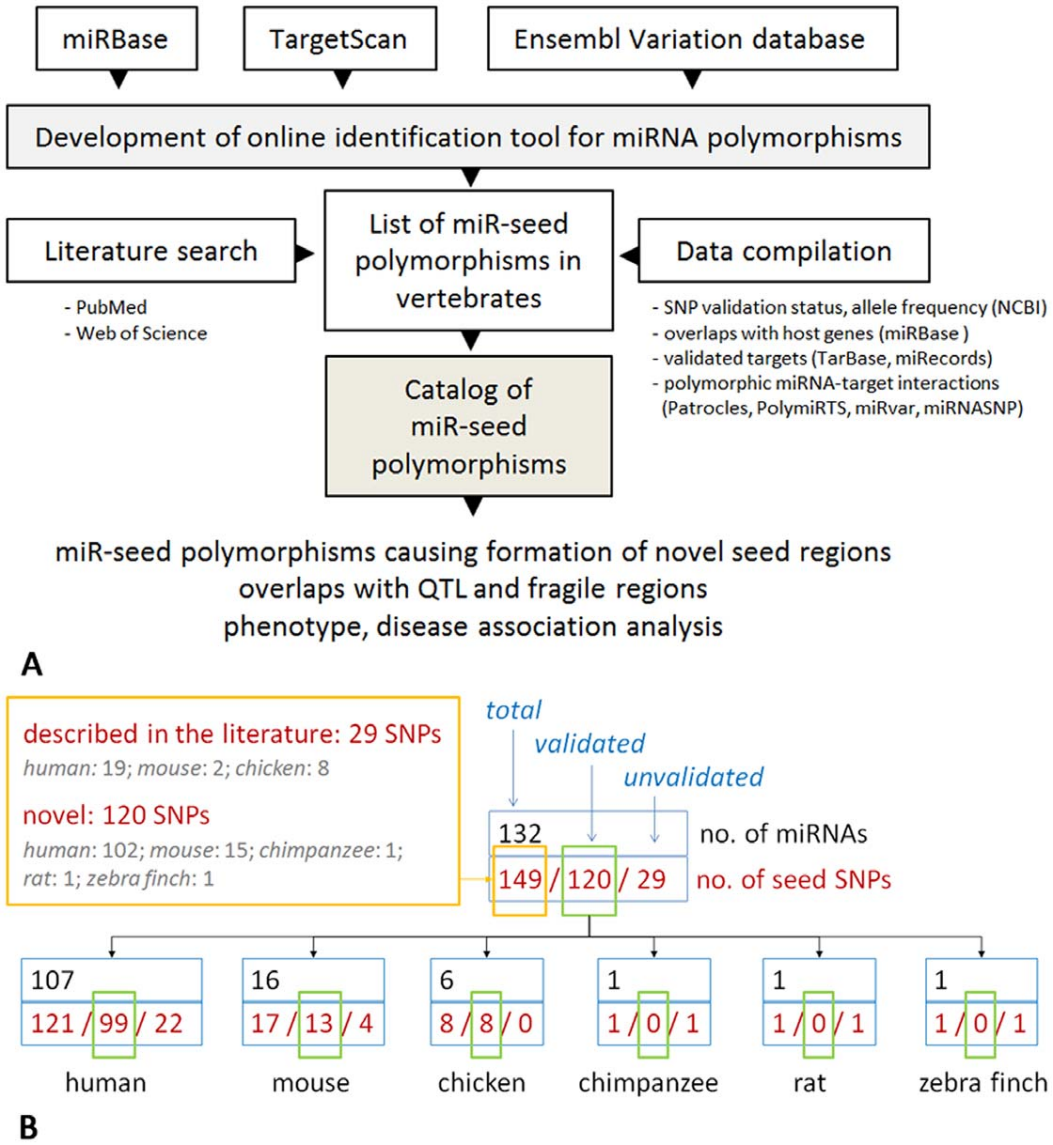
Workflow diagram of the study and diagram of assembled polymorphic miRNAs. (**A**) Workflow diagram of the study: approaches applied for search of known and novel seed miRNA variations and further bioinformatic analysis performed on the database of miR-seed polymorphisms. (**B**) Diagram of assembled miRNAs comprising miR-seed-SNPs according to source, validation status and species.

We developed a web-based tool for the detection of genetic variations in miRNA genes in vertebrates and generated an open access catalog of polymorphisms that overlap with miRNA seed regions (**Fig. 1A**). This catalog was supplemented with information relevant for further functional analysis. Genotype-phenotype analysis of the murine miR-seed-SNP located in *mmu-miR-717* showed association with a diverse array of traits.

### Development of the online tool for the detection miRNA gene polymorphisms

**Figure 2:**
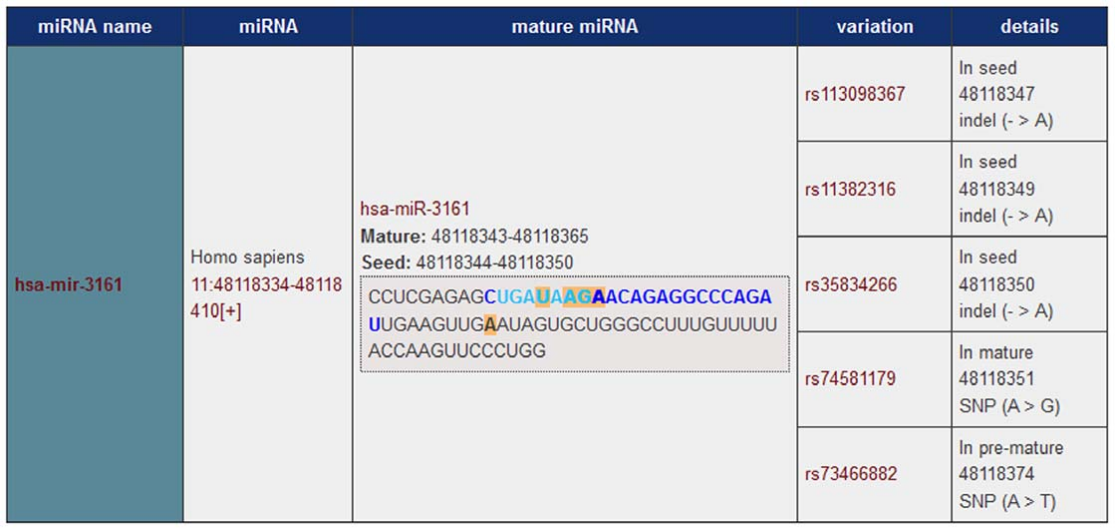
Output of developed miRNA SNiPer tool. An example of miR-SNPs located in pre-miRNA, mature, or seed region of the human miRNA *hsa-miR-3161*. Mature miRNA sequence are highlighted in dark blue, seed regions in light blue, and polymorphisms in orange.

The online tool miRNA SNiPer for the detection of genetic polymorphisms residing within miRNA genes in vertebrates (http://www.integratomics-time.com/miRNA-SNiPer/) was developed using data assembled from miRBase, TargetScan and Ensembl Variation database (**Fig. 2**). The search for miR-seed SNPs was performed in thirteen vertebrate species: chicken, chimpanzee, dog, horse, human, mouse, opossum, pig, platypus, pufferfish, rat, zebra finch, and zebrafish.

Display settings enable the miR-SNPs to be arranged according to their location in pre-miRNAs, mature or seed regions. In six vertebrate species (human, mouse, chicken, chimpanzee, rat, and zebra finch) 149 polymorphisms overlapped with miRNA seed regions (**Fig. 1B** **, Table S1**). These polymorphisms included SNPs, double nucleotide polymorphisms (DNPs), and insertion/deletions (indels). An example of miR-SNPs located within the pre-miRNA, mature and seed regions of miRNA gene is demonstrated in **Figure 2**.

Data from species in which the latest releases of Ensembl Variation Database and miRBase assemblies are currently not compatible were not included in the catalog (cat, cattle, rabbit, etc.). The miRNA SNiPer tool is going to be updated with each new release of compatible databases.

### Catalog and genomic view of miR-seed polymorphisms in vertebrates

**Figure 3:**
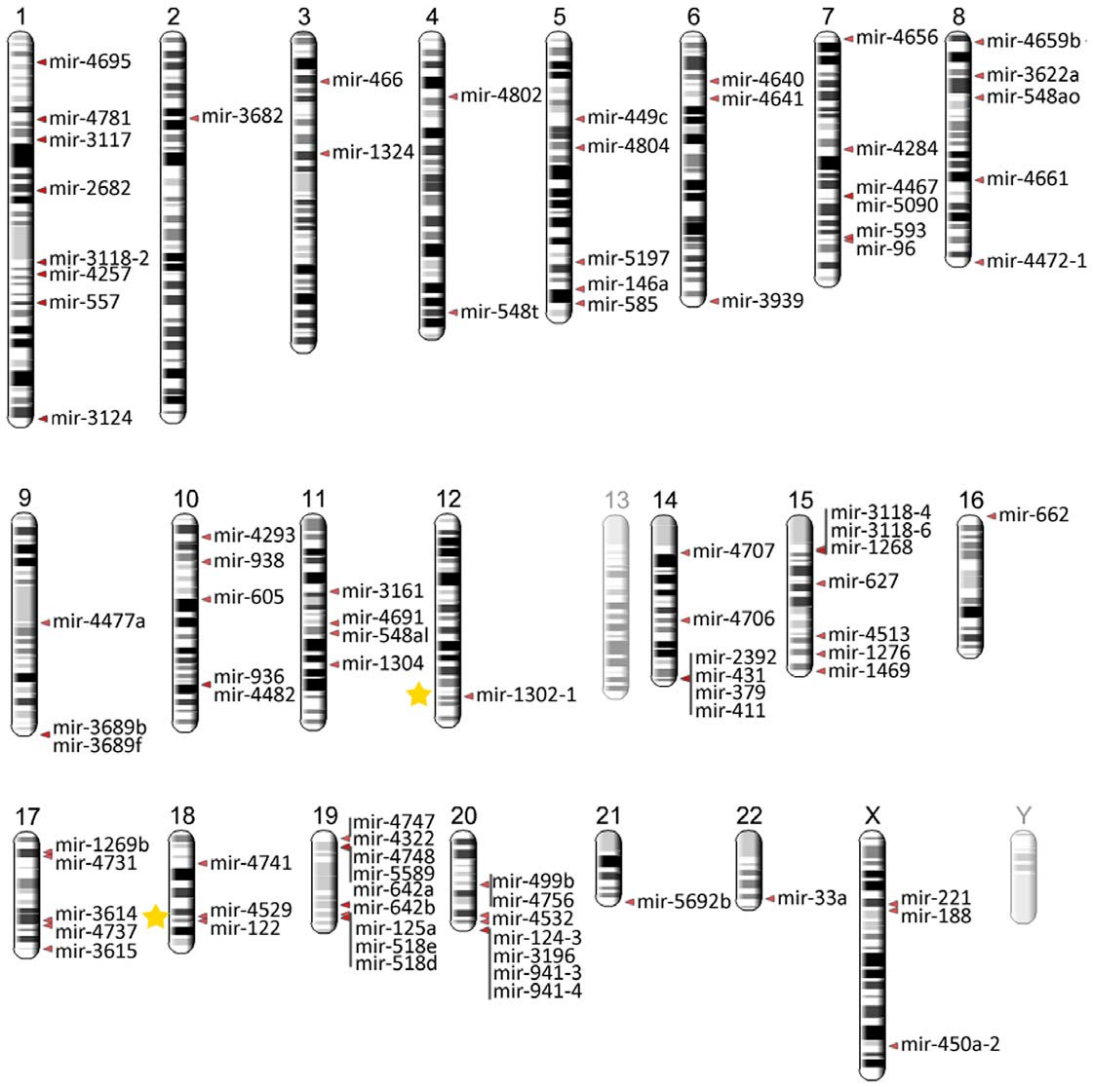
Genomic location of miRNAs with polymorphic seed regions in human. miRNAs comprising validated seed region polymorphisms mapping to two overlapping fragile sites are marked with yellow stars.

The list of miR-seed polymorphisms in six species (human, mouse, chicken, chimpanzee, rat, and zebra finch) was supplemented with data retrieved from literature and databases, and presented as an open access online catalog (http://www.integratomics-time.com/miR-seed-SNPs/catalog/). From the total of 149 identified miR-seed polymorphisms only 29 have previously been described in the miRNA context [8]–[10], [12], [13], [32]–[45] (**Fig. 1B**). Among them four studies discussed the miR-seed-SNPs as located in the mature region of the miRNA without referring to their miRNA seed location [36], [39], [44], [45]. The remaining 120 miR-seed polymorphisms from the catalog have not been described in publications previously. Data integration revealed that 120 of 149 miR-seed polymorphisms had been previously validated or genotyped. The catalog was additionally supplemented with information of the host gene location and orientation, validation status of miRNA target genes and SNPs (**Table S1**). Genomic distribution of the assembled miR-seed-SNPs was presented on the genomic view (http://www.integratomics-time.com/miR-seed-SNPs/genomic_view) (**Fig. 3**).

The frequency estimations of miRNAs having polymorphic seed regions at this point should be treated with caution because all miRNAs have not yet been systematically sequenced and screened for polymorphisms, and some SNPs in the databases still have unvalidated status. Our preliminary data showed that in human 88 of 1527 (5.7%) currently annotated miRNAs had polymorphic seed regions (99 validated seed-SNPs in total). Similarly, in mouse 13 of 741 (1.7%) miRNAs had miR-seed-SNPs (all previously validated), whereas in chicken six of 499 (1.2%) miRNAs had miR-seed-SNPs (eight validated). Nevertheless, our analysis of currently available data revealed a much higher frequency (5.9%) of miR-seed polymorphisms in human than the frequency of >1% reported by Didiano and Hobert [46]. Similarly, Muiños-Gimeno *et al.* [47] also revealed much lower frequencies than our study mapping 24 SNPs within 325 mature miRNAs and detecting only two miR-seed-SNPs. They also estimated the density of SNPs in miRNAs to be 4.5-times lower that in the rest of the genome [47]. In contrast to this, we found that some miRNAs had highly polymorphic seed regions. For instance, pre-miRNA *gga-mir-1658* had one SNP within the mature sequence (*gga-miR-1658*) and two SNPs within the minor miR sequence (gga-miR-1658*). Our study also revealed four human miRNAs (*hsa-miR-96*, -*518e-5p*, -*1304-5p*, and -*3939*) that had two validated consecutive nucleotides altered, so called DNPs, which have been found to occur with a frequency of ∼1% of the total number of SNPs in the genome [48]. It has been suggested that DNPs have a greater propensity to be involved in disease causing mutations in protein coding regions as they effect two positions in a codon, resulting in a more likely non-synonymous mutation [48]. We can speculate that miR-seed-DNPs identified in our study (four validated and five unvalidated) also have a potential to cause more profound effects on the regulation of target genes and phenotypes that single miR-seed-SNPs, but this is to be evaluated experimentally in future studies.

Allele frequency was available for 90 SNPs in human, 12 in mouse, and eight SNPs in chicken (NCBI). Population-based differences were observed for 41 human SNPs; among them rs12975333 described as polymorphic in three studies [9], [32], [35], but monomorphic in the Spanish [47] and Scandinavian populations [49]. As expected, transitions (purine ↔ purine or pyrimidine ↔ pyrimidine substitutions) were twice as more frequent than transversions (purine ↔ pyrimidine). Because transversions induce greater genetic alternations that are more likely to cause functional consequences [50] we have examined their prevalence in miR-seed-SNPs and found 31 transversions; 27 in human, three in mouse, and one in chicken. Interestingly, transversions were observed in *hsa-miR-96* and *mmu-miR-96* which have been previously linked with hearing loss in both human and mouse [10], [12].

### Data analysis

The data from the catalog were further analyzed to prioritize promising SNPs for further functional analysis. We examined each miR-seed SNP for their potential to generate novel seed regions that also match to a different miRNA, for their genomic distribution, overlaps with host genes, QTL, and fragile regions, as well as their association with diseases and phenotypes.

#### miR-seed polymorphisms causing the formation of a seed region annotated to a different miRNA

As shown in Mencia *et al.*, [10] a miR-seed-SNP+13G>A in *hsa-miR-96* caused a change in the seed region to perfectly match another annotated miRNA *hsa-miR-514*, implying a possibility of targets shared by both miRNAs. To determine whether miR-seed-SNPs cause the formation of seed regions annotated to different miRNAs we screened the catalog using TargetScan Custom. SNPs in *hsa-miR-3117-3p* and *-4467* matched two different seed regions of *hsa-miR-499-5p* and *-885-3p*, respectively (**Figure S1**). A change of annotated seed regions may lead to altered recognition and selection of miRNA targets, which could possibly be a part of a different biological pathway.

#### Genomic distribution of miR-seed-SNPs and their overlaps with host genes, QTL, and fragile regions

Genomic locations of miRNAs comprising seed polymorphisms are shown in **Figure 3**, **Figures S2** and **S3** for human, mouse, and chicken, respectively. The highest number of miR-seed polymorphisms was present on human chromosomes 1, 15, 19, and 20. Several miRNAs with polymorphic seed regions overlapped with host genes, QTL, fragile sites, or cancer susceptibility sites.

We observed 75 miRNAs with polymorphic seed regions residing within protein coding host genes; 16 in antisense, 55 in sense orientation and four miRNAs that overlapped with host genes both orientations (**Table S1**). MiRNA genes and their host genes in antisense orientation have been shown to have independent transcription mechanisms [51], whereas sense transcriptional orientation suggests that miRNAs and host genes can be transcribed from shared promoters [1]. Sense oriented miRNA genes from our catalog were either exonic (five in human and three in mouse), intronic (34 in human, three in mouse, and three in chicken), or overlapped both exonic and intronic transcripts (seven in human) (**Table S1**). Intronic miRNAs have previously been found to be co-expressed and regulated by co-activation of both miRNA and its host gene [52], [53]. Several studies have also shown that host genes are functionally linked with their resident miRNAs [53]–[55].

By comparing locations of polymorphic seed regions with QTL and fragile sites, several overlaps were found. MiRNAs with validated seed SNPs overlapped with 830 QTL in human, 118 in mouse and 20 in chicken. Highest number of overlapped QTL in human was observed for *hsa-miR-4737* and *hsa-miR-4756* each overlapping 43 QTL. In mouse mmu-miR-628 overlapped with 23 QTL and in chicken gga-miR-1658 with seven QTL (data not shown). MiRNA genes have also been observed to be frequently located near the mouse cancer susceptibility loci, which is in concordance with a previous study of Sevignani *et al.* [56]. These results support previous observations that miRNAs are an important player in generating genetic variability and important genomic sites in the trait's genetic architecture.

*Hsa-miR-1302-1* overlapped with a common fragile site located at 12q24.1 and *hsa-miR-122-3p* overlapped with a fragile site located at 18q21.3 (**Fig. 3**). *Hsa-miR-513a-5p* overlapped with a fragile site located at cytogenetic band Xq27; however, this SNP is yet to be validated. This observation is in concordance with a previous study showing that miRNAs are frequently located at fragile sites, as well as minimal regions of loss of heterozygosity, minimal regions of amplification, or common breakpoint regions [57].

#### MiR-seed-SNP association with diseases and phenotypes

We reviewed published associations between miRNAs with polymorphic seed regions and diseases/phenotypes. Additionally, we performed a statistical genotype-phenotype association analysis using the data from the Mouse Phenome Database. Four human and two mouse miRNAs comprising seed-SNPs have already been associated with diseases and phenotypes (**Table 1**) [10], [12], [13], [32]–[34], [37]–[39], [41], [42], [44], [45], [58]. Hsa-miR-146a-3p and -499-3p were associated with the largest variety of pathologies affecting all organ systems, especially the reproductive and digestive system. Two separate studies have linked the SNP in the seed region of miR-96 to the same clinical pathology, hearing loss, in mouse [12] and human [10], which also represents the first case implicating a miRNA in a Mendelian inherited disorder. In a recent study, Kunej *et al.* [13] analyzed a murine SNP (rs30372501) in *mmu-miR-717* seed region which was found to be associated with leanness. Evidence also exists that miR-717 is involved in osmoregularity control and is regulated rapidly in response to high salt exposure in mice [59]. To verify if these associations hold true for some other standard inbred mouse strains we searched for association between the *mmu-miR-717* SNP (rs30372501) genotypes and all 2586 phenotypes within the Mouse Phenome Database (see Material and Methods). The SNP rs30372501 showed a significant effect (p<0.01) on 363 measurements-parameters that are grouped by MPD into 25 trait-groups: 23 in behavior, 27 in blood-clinical chemistry, 40 in blood-hematology, 22 in blood-lipids, one in blood-miscellaneous, 19 in body composition, 38 in body weight size and growth, 50 in bone, one in brain, two in breathing pattern, one in cancer, five in cardiovascular, three in cell and tissue damage, five in ear, four in endocrine, two in gallbladder, 18 in immune system, three in ingestive preference, 11 in kidney, four in liver, seven in local experiment parameter, nine in muscle, 23 in respiratory, one in sensory gating, two in spleen, and 42 in ungrouped **(Table S2)**. A result that seed SNP rs30372501 was significantly associated with 363 measurements-parameters should be closely examined and interpreted further. As shown in **Table S2**, many measurements within a group are highly correlated (e.g. body weights at various ages within a group “body weight size and growth”) and also groups of traits can be highly correlated (e.g., body weight and body composition traits, fat depot weight etc.). Therefore, one should not interpret these associations as causal but rather as a list of potential groups of traits that a particular miR-SNP could affect. In this sense a visual presentation (**Figure S4**) of associations shown in **Table S2** can be informative as one can observe immediately which groups have the highest number of significant associations and hence string support and which trait groups can be further joined into related “super” groups (e.g. body weight, body composition, blood lipids etc.). Such examination can help researchers to prioritize further causation experiments by providing them only a small number of different traits likely to be controlled by a miR-SNP.

**Table 1:** Diseases and phenotypes associated with miRNA gene polymorphisms within the seed region in human and mouse.

miRNA	miRNA genomic location	Associated diseases and phenotypes
**HUMAN**		
**hsa-miR-96**	chr 7: 129414532–129414609	nonsyndromic progressive hearing loss [10]
**hsa-miR-125a-5p**	chr 19: 52196507–52196592	breast cancer [32]
**hsa-miR-146a-3p**	chr 5: 159912359–159912457	breast and ovarian cancer [33]; hepatocellular carcinoma [34]; thyroid cancer [37]; esophageal squamous cell carcinoma [38]; gastric cancer [39]; dilated cardiomyopathy [58]; prostate cancer [41]; cervical squamous cell carcinoma [45]
**hsa-miR-499a-3p**	chr 20: 33578179–33578300	breast cancer [36]; gastric cancer [39]; prostate cancer [42]; ulcerative colitis [44]; cervical squamous cell carcinoma [45]
**MOUSE**		
**mmu-miR-96**	chr 6: 30119446–30119551	hearing loss [12]
**mmu-miR-717**	chr X: 49775584–49775692	leanness [13]; behavior, blood-clinical chemistry, blood-hematology, blood-lipids, blood-miscellaneous, body composition, body weight size and growth, bone, brain, breathing pattern, cancer, cardiovascular, cell and tissue damage, ear, endocrine, gallbladder, immune system, ingestive preference, kidney, liver, local experiment parameter, muscle, respiratory, sensory gating, spleen (present study)

**Figure 4:**
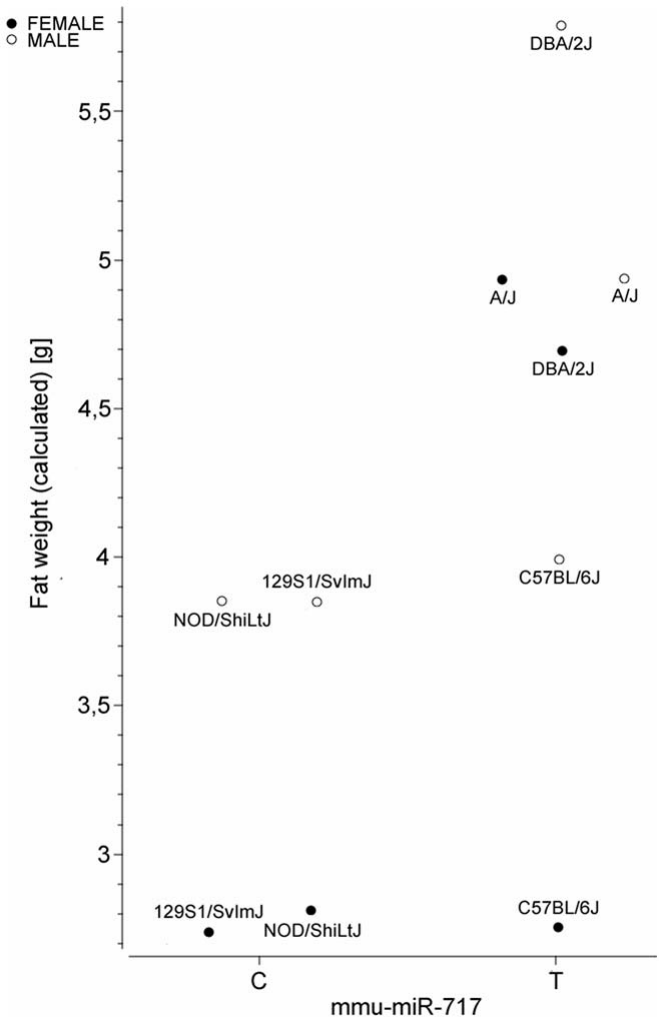
MiR-seed-SNP within *mmu-miR-717* is associated with fat weight in mice. Association analysis between *mmu-miR-717* SNP genotypes in different inbred mouse strains and phenotypes within the Mouse phenome database revealed that *mmu-miR-717* significantly affects several different traits. An example of significant difference between lean and high fat strains that differ for *mmu-miR-717* SNP genotype (C>T) and fat weight, for both female and male is shown.

Associations between SNP rs30372501 and obesity traits reported by Kunej *et al.* [13] were confirmed also in the present statistical analysis. **Figure 4** shows significant differences in Fat weight (g) between the lean mouse strains carrying a seed SNP rs30372501 allele C (e.g., 129S1/SvImJ, NOD/ShiLtJ) and allele T-carrying high fat strains (e.g., A/J, DBA/2J) in both sexes. Such miR-SNP-genotype to phenotype association analyses can help researchers select an optimal strain and phenotype for further experiments as well as identifying traits and pathologies likely to be affected by miR-SNP variability.

### Future perspectives

The following open questions could be addressed in future studies: 1) To examine the effects of SNPs that cause a formation of seed regions already annotated to different miRNAs. 2) To experimentally validate DNPs found in seed regions for their effect on miRNA target selection. 3) To study effects of miR-seed-SNPs identified herein on shared transcriptional regulation, expression and function of polymorphic miRNAs and their host genes. 4) Our statistical association analysis of seed-SNP with mouse phenotypes showed a diverse array for associated phenotypes. Further studies should be designed to examine the molecular mechanism for such differential miR-SNP pleiotropic effects.

In conclusion, miR-seed polymorphisms may have a profound effect on a wide range of phenotypes. Using the database integration we assembled all known and identified novel miR-seed-SNPs, and performed a first systematic case-study in this field. The project is ongoing, as novel miRNAs and SNPs are yet to be discovered in human as well as in other animal species. However, results and tools developed in this study can be immediately used by interested scientific community to help retrieve valuable information and design efficient experimental plans in the field of miR-SNP research.

## Supporting Information

Figure S1 miR-seed-SNP causing formation of novel seed regions. Three examples of miRNAs (green) with seed-SNPs which cause a formation of a seed region annotated to another miRNA are indicated (red).
(TIF)

Figure S2 Genomic location of miRNAs with polymorphic seed regions in mouse.
(TIF)

Figure S3 Genomic location of miRNAs comprising seed polymorphisms in chicken.
(TIF)

Figure S4 Graphical representation of Table S2 showing association between *mmu-miR-717* seed SNP rs30372501 and 363 traits clustered into 25 groups: behavior, blood-clinical chemistry, blood-hematology, blood-lipids, blood-miscellaneous, body composition, body weight size and growth, bone, brain, breathing pattern, cancer, cardiovascular, cell and tissue damage, ear, endocrine, gallbladder, immune system, ingestive preference, kidney, liver, local experiment parameter, muscle, respiratory, sensory gating and spleen.
(TIF)

Table S1 Catalog of miRNAs with polymorphic seed regions in human, mouse, chicken, chimpanzee, rat, and zebra finch: genomic location, host gene orientation, nucleotide substitution and validation status of the SNP.
(DOC)

Table S2 Estimated differences between miR-seed- SNP (rs30372501) alleles (C>T), associated standard errors and P_values for 363 traits.
(DOC)
