# Supplementary figures and images for: Catalog of MicroRNA Seed Polymorphisms in Vertebrates
Source: PLoS One. 2012 Jan 27;7(1):e30737. doi: 10.1371/journal.pone.0030737 (PMC3267754; doi:10.1371/journal.pone.0030737)

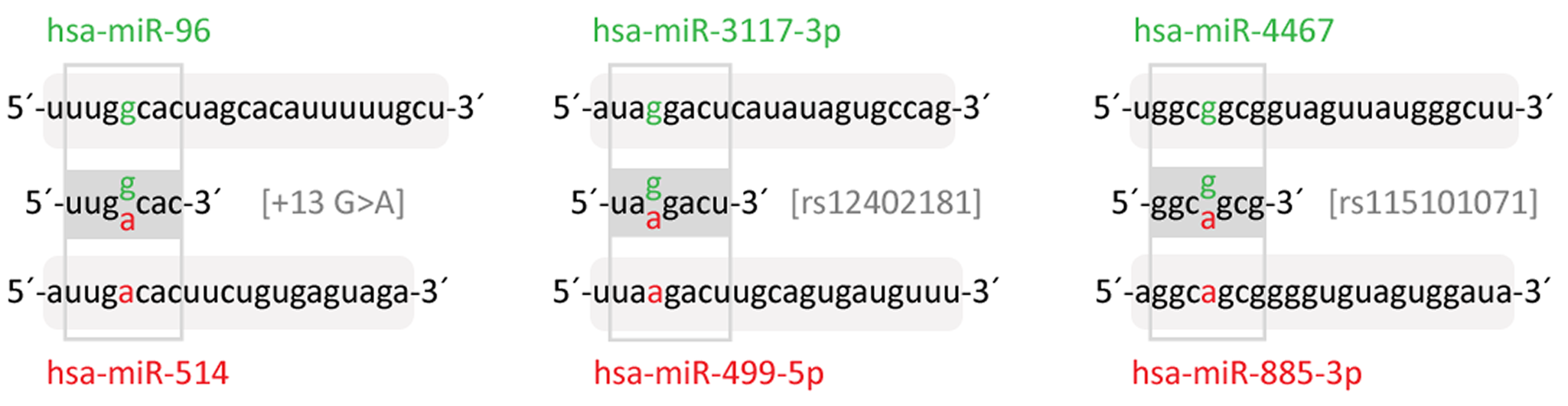

Supplement: Figure S1 — miR-seed-SNP causing formation of novel seed regions. Three examples of miRNAs (green) with seed-SNPs which cause a formation of a seed region annotated to another miRNA are indicated (red). (TIF) [file pone.0030737.s001.tif]

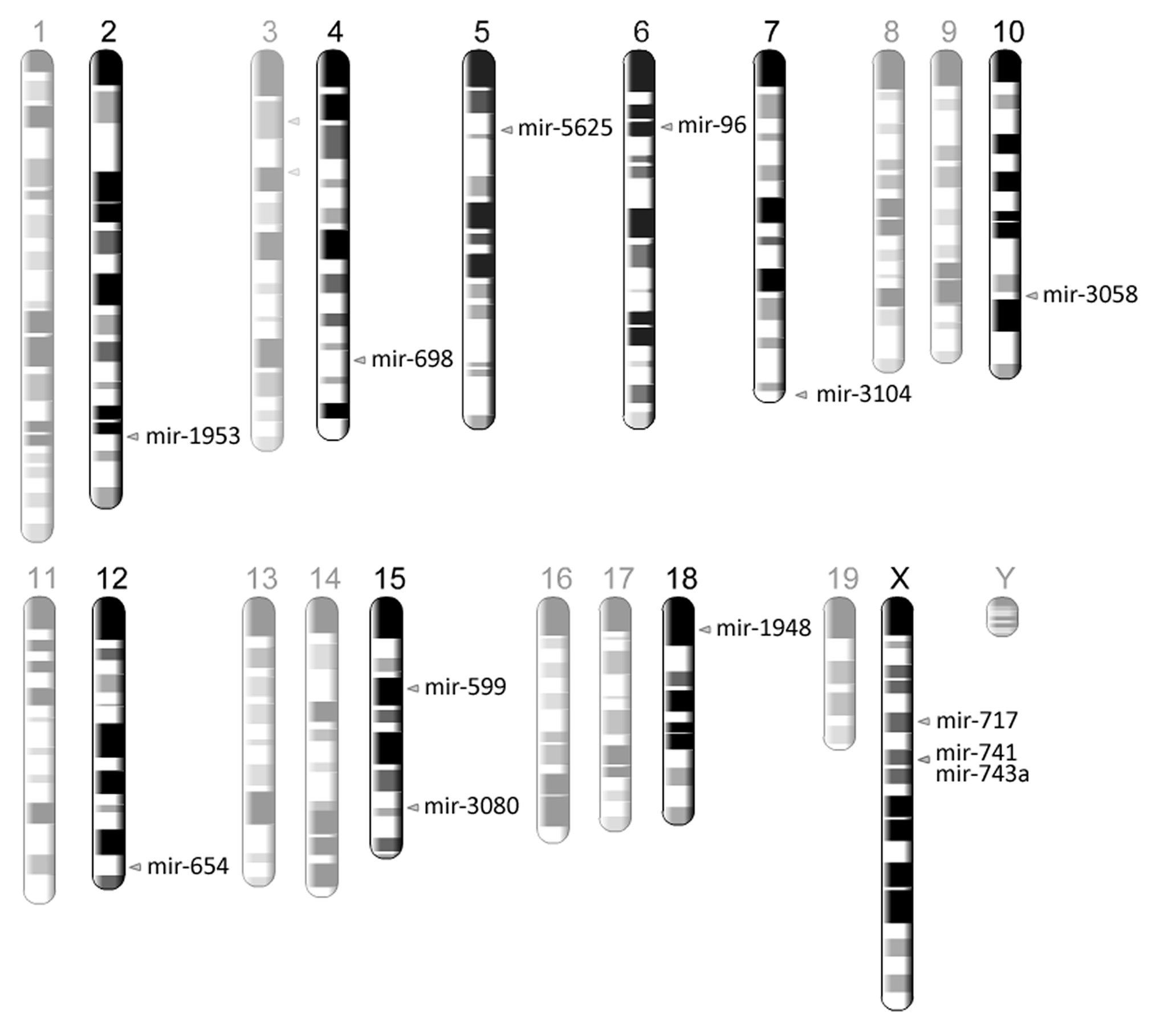

Supplement: Figure S2 — Genomic location of miRNAs with polymorphic seed regions in mouse. (TIF) [file pone.0030737.s002.tif]

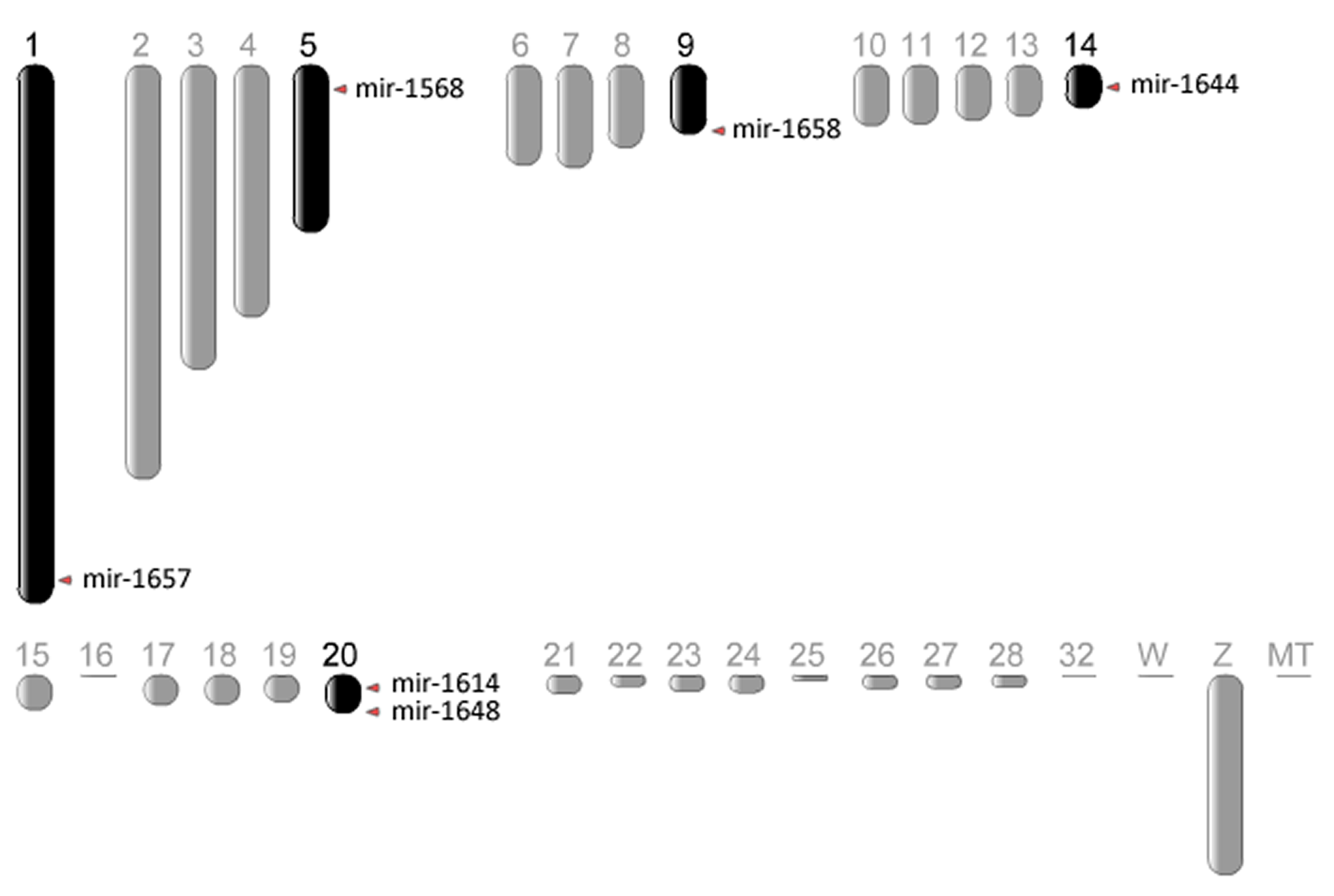

Supplement: Figure S3 — Genomic location of miRNAs comprising seed polymorphisms in chicken. (TIF) [file pone.0030737.s003.tif]

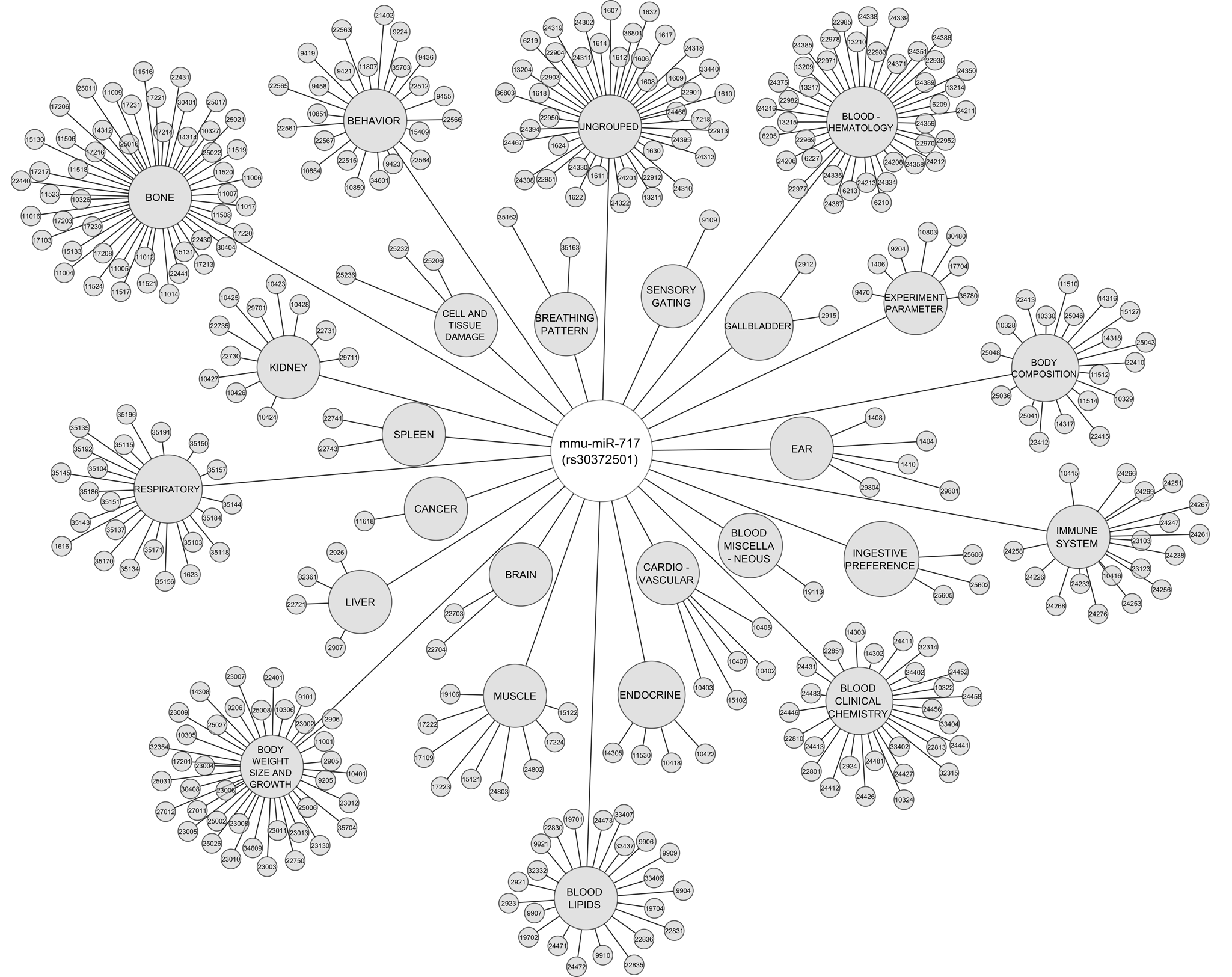

Supplement: Figure S4 — Graphical representation of Table S2 showing association between mmu-miR-717 seed SNP rs30372501 and 363 traits clustered into 25 groups: behavior, blood-clinical chemistry, blood-hematology, blood-lipids, blood-miscellaneous, body composition, body weight size and growth, bone, brain, breathing pattern, cancer, cardiovascular, cell and tissue damage, ear, endocrine, gallbladder, immune system, ingestive preference, kidney, liver, local experiment parameter, muscle, respiratory, sensory gating and spleen. (TIF) [file pone.0030737.s004.tif]
