# Supplementary material for: Catalog of MicroRNA Seed Polymorphisms in Vertebrates
Source: PLoS One. 2012 Jan 27;7(1):e30737. doi: 10.1371/journal.pone.0030737 (PMC3267754; doi:10.1371/journal.pone.0030737)
Supplement: Table S1 — Catalog of miRNAs with polymorphic seed regions in human, mouse, chicken, chimpanzee, rat, and zebra finch: genomic location, host gene orientation, nucleotide substitution and validation status of the SNP. (DOC) [file pone.0030737.s005.doc]

# Table S1. Catalog of miRNAs with polymorphic seed regions in human, mouse, chicken, chimpanzee, rat, and zebra finch: genomic location, host gene orientation, nucleotide substitution and validation status of the SNP.

| **miRNA name** | **miRNA location/ host gene ^1^** | **host gene /miRNA orientation** | **validated miRNA targets** | **SNP ID^2^** | **nucleotide substitution** | **identification/ validation/ genotyping [reference]** |
| --- | --- | --- | --- | --- | --- | --- |
| ***Validated polymorphisms*** | | | | | | |
| **HUMAN** | | | | | | |
| **hsa-miR-33a-3p** | *SREBF2* | intronic/s | *ABCA1* | rs77809319 | A>G | NCBI |
| **hsa-miR-96** | intergenic | / | (10 targets) | **+13 G>A** | G>A | [10] |
|  |  |  |  | **+14 C>A** | C>A | [10] |
| **hsa-miR-122-3p** | intergenic | / | (50 targets) | rs41292412 | C>T | [35], NCBI |
| **hsa-miR-124-3-3p** | *RP5-963E22.4* | intronic/as | (206 targets) | rs34059726 | G>T | [35] |
| **hsa-miR-125a-5p** | *AC018755.2* | exonic/as | (6 targets) | rs12975333 | G>T | [9,32],[35] |
| **hsa-miR-146a-3p** | *CTC-231O11.1* | exonic/s | (34 targets) | rs2910164 | C>G | [33,34,37-39,41,43,45,58], NCBI |
| **hsa-miR-188-3p** | *CLCN5* | intronic/s | - | 3p 60: C>T | C>T | [8] |
| **hsa-miR-221-5p** | intergenic | / | (21 targets) | rs113054794 | C>A | NCBI |
| **hsa-miR-379-3p** | intergenic | / | - | rs61991156 | A>G | [35] |
| **hsa-miR-411-3p** | intergenic | / | - | rs111835650 | T>C | NCBI |
| **hsa-miR-431-3p** | *RTL1* | exonic/as | - | rs12884005 | G>A | [35] |
| **hsa-miR-449c-3p** | *CDC20B* | intronic/s | - | rs35770269 | A>T | NCBI |
| **hsa-miR-450a-2** | intergenic | / | - | 5p 4: T>C | T>C | [8] |
| **hsa-miR-466** | intergenic | / | - | rs116476604 | A>G | NCBI |
| **hsa-miR-499a-3p** | *MYH7B* | intronic/s | - | rs3746444 | A>G | [35,36,39,42,44,45], NCBI |
| **hsa-miR-518d-3p** | intergenic | / | - | rs73602910 | G>A | NCBI |
| **hsa-miR-518e-5p** | intergenic | / | - | rs74177813 | C>- | NCBI |
|  |  |  |  | **rs34416818** | ->A | [35] |
|  |  |  |  | **rs74177814** | ->G | NCBI |
| **hsa-miR-548al** | *RP11-702H23.4* | intronic/s | - | rs515924 | A>G | NCBI |
| **hsa-miR-548ao-3p** | *SFRP1* | intronic/s | - | rs79091838 | G>A | NCBI |
| **hsa-miR-548t-3p** | *GALNT7* | intronic/s | - | rs73872515 | A>C | NCBI |
|  | *AC097534.1* | exonic/as |  |  |  |  |
| **hsa-miR-557** | intergenic | / | - | rs78825966 | C>T | NCBI |
| **hsa-miR-585** | *SLIT3* | intronic/s | - | rs62376935 | C>T | [35], NCBI |
| **hsa-miR-593-5p** | *SND1* | intronic/s | - | rs73721294 | C>T | NCBI |
| **hsa-miR-605** | *PRKG1* | intronic/s | *SEC24D* | [rs113212828](http://www.ncbi.nlm.nih.gov/sites/entrez?db=snp&cmd=search&term=rs113212828) | A>G | NCBI |
| **hsa-miR-627** | *VPS39* | intronic/s | - | rs2620381 | T>G | [9,35], NCBI |
| **hsa-miR-642a-5p** | *GIPR* | intronic/s | - | rs78902025 | T>G | NCBI |
| **hsa-miR-642b-3p** | *MIR642A* | exonic/as | - | rs111664333 | G>A | NCBI |
|  | *GIPR* | intronic/as |  |  |  |  |
| **hsa-miR-662** | *MSLNL* | exonic/as | - | rs9745376 | G>A | [9,35], NCBI |
| **hsa-miR-936** | intergenic | / | - | rs79924817 | T>C | NCBI |
| **hsa-miR-938** | *SVIL* | intronic/s | - | rs12416605 | G>A | [35], NCBI |
| **hsa-miR-941-3** | *DNAJC5* | intronic/s | - | rs113672516 | A>G | NCBI |
|  |  |  |  | rs35544770 | A>G | [35], NCBI |
| **hsa-miR-941-4** | *DNAJC5* | intronic/s | - | rs35544770 | A>G | NCBI |
| **hsa-miR-1268a** | intergenic | / | - | rs28599926 | C>T | [35], NCBI |
| **hsa-miR-1269b** | *ARHGAP44* | intronic/as | - | rs7210937 | G>C | NCBI |
|  | *AC005277.1* | intronic/as |  |  |  |  |
| **hsa-miR-1276** | *KLHL25* | intronic/s | - | rs34381260 | ->A | [35] |
| **hsa-miR-1302-1** | *RPH3A* | intronic/as | - | rs74647838 | G>A | NCBI |
| **hsa-miR-1304-5p** | *TAF1D* | ex./int./s | - | **rs76857625** | A>G | NCBI |
|  |  |  |  | **rs79759099** | A>G | NCBI |
| **hsa-miR-1324** | intergenic | / | - | rs111606583 | C>T | NCBI |
|  |  |  |  | rs113859132 | G>A | NCBI |
| **hsa-miR-1469** | *NR2F2* | intronic/s | - | rs116596918 | G>C | NCBI |
| **hsa-miR-2392** | *MEG3* | intronic/s | - | rs118055959 | A>G | NCBI |
| **hsa-miR-2682-3p** | *RP4-672J20.2* | exonic/s | - | rs74904371 | C>T | NCBI |
|  | *RP11-490G2.1* | intronic/s |  |  |  |  |
| **hsa-miR-3117-3p** | *SGIP1* | intronic/s | - | rs12402181 | G>A | NCBI |
| **hsa-miR-3118-2** | *BX571672.1* | intronic/s | - | **rs61786895** | G>A | NCBI |
|  | *BX571672.4* | intronic/s |  |  |  |  |
|  | *BX571672.2* | intronic/as |  |  |  |  |
| **hsa-miR-3118-4** | intergenic | / | - | rs7167371 | C>G | NCBI |
| **hsa-miR-3118-6** | intergenic | / | - | rs7167371 | C>G | NCBI |
| **hsa-miR-3124-5p** | *SH3BP5L* | exonic/as | - | rs12081872 | C>T | NCBI |
| **hsa-miR-3161** | *PTPRJ* | intronic/s | - | rs113098367 | ->A | NCBI |
|  |  |  |  | **rs11382316** | ->A | NCBI |
| **hsa-miR-3196** | *BIRC7* | intronic/s | - | rs113297757 | G>A | NCBI |
| **hsa-miR-3614-5p** | *TRIM25* | exonic/s | - | rs118080115 | T>C | NCBI |
| **hsa-miR-3615** | *SLC9A3R1* | exonic/s | - | rs112977728 | C>T | NCBI |
| **hsa-miR-3622a-5p** | intergenic | / | - | rs66683138 | G>A | NCBI |
| **hsa-miR-3682-5p** | *ASB3* | intronic/s | - | rs116380885 | T>C | NCBI |
| **hsa-miR-3689b-5p** | intergenic | / | - | rs116838571 | T>A | NCBI |
| **hsa-miR-3689f** | intergenic | / | - | rs73664170 | T>C | NCBI |
| **hsa-miR-3939** | *RP1-167A14.2* | intronic/s | - | rs76608449 | G>C | NCBI |
|  |  |  |  | **rs75823810** | C>T | NCBI |
|  |  |  |  | **rs73024232** | G>A | NCBI |
| **hsa-miR-4257** | *ADAMTSL4* | intronic/s | - | rs74743733 | G>A | NCBI |
|  | *RP11-54A4.2* | intronic/as |  |  |  |  |
| **hsa-miR-4284** | *STX1A* | intronic/as | - | rs11973069 | C>T | NCBI |
| **hsa-miR-4293** | *RP11-397C18.4* | intronic/s | - | rs12220909 | G>C | NCBI |
|  | *FRMD4A* | intronic/s |  |  |  |  |
| **hsa-miR-4322** | *S1PR2* | intronic/as | - | rs114399468 | G>A | NCBI |
| **hsa-miR-4467** | *LRWD1* | ex./int./s | - | rs115101071 | G>A | NCBI |
|  |  |  |  | rs76625393 | C>T | NCBI |
| **hsa-miR-4472-1** | intergenic | / | - | rs28655823 | G>C | NCBI |
| **hsa-miR-4477a** | intergenic | / | - | rs34026740 | C>T | NCBI |
|  |  |  |  | rs75019967 | A>C | NCBI |
| **hsa-miR-4482-1** | intergenic | / | - | rs45596840 | A>G | NCBI |
| **hsa-miR-4513** | *CSK* | intronic/as | - | rs2168518 | C>T | NCBI |
| **hsa-miR-4529-3p** | *TCF4* | intronic/as | - | rs111734691 | G>A | NCBI |
| **hsa-miR-4532** | intergenic | / | - | rs113808830 | C>T | NCBI |
|  |  |  |  | rs60432575 | G>A | NCBI |
| **hsa-miR-4640-3p** | *DDR1* | ex./int./s | - | rs61596908 | ->CT | NCBI |
| **hsa-miR-4641** | *FOXP4* | intronic/s | - | rs77674981 | G>A | NCBI |
| **hsa-miR-4656** | *AC092610.9* | exonic/as | - | rs71535143 | C>- | NCBI |
|  | *KIAA0415* | intronic/as |  |  |  |  |
| **hsa-miR-4659b-5p** | *AGPAT5* | intronic/as | - | rs74601816 | C>A | NCBI |
| **hsa-miR-4661-3p** | *LRRC69* | intronic/s | - | rs12335005 | G>T | NCBI |
| **hsa-miR-4691-3p** | *NDUFS8* | ex./int./s | - | rs115430932 | C>T | NCBI |
| **hsa-miR-4695-5p** | *ALDH4A1* | intronic/s | - | rs79637190 | C>T | NCBI |
|  | *RP13-279N23.2* | intronic/s |  |  |  |  |
| **hsa-miR-4706** | *FNTB* | intronic/s | - | rs2296320 | C>T | NCBI |
|  | *MAX* | intronic/as | - | rs72728267 | G>T | NCBI |
|  | *RP11-840I19.2* | intronic/s |  |  |  |  |
| **hsa-miR-4707-3p** | *HAUS4* | exonic/s | - | rs2273626 | A>C | NCBI |
|  | *RP11-298I3.5* | intronic/s |  |  |  |  |
| **hsa-miR-4731-3p** | *PMP22* | intronic/s | - | rs66507245 | T>A | NCBI |
| **hsa-miR-4737** | intergenic | / | - | rs111365058 | A>G | NCBI |
| **hsa-miR-4741** | *RBBP8* | exonic/s | - | rs7227168 | C>T | NCBI |
|  |  |  |  | rs115479920 | G>A | NCBI |
| **hsa-miR-4747-3p** | *UHRF1* | intronic/s | - | rs77046863 | C>T | NCBI |
| **hsa-miR-4748** | *DNM2* | intronic/s | - | rs76796065 | G>T | NCBI |
| **hsa-miR-4756-3p** | *BCAS1* | intronic/s | - | rs209426 | A>T | NCBI |
| **hsa-miR-4781-3p** | *TCEANC2* | intronic/s | - | rs74085143 | G>A | NCBI |
| **hsa-miR-4802-5p** | *RBM47* | intronic/s | - | rs112628148 | T>C | NCBI |
| **hsa-miR-4804-5p** | *TNPO1* | intronic/s | - | rs266435 | C>G | NCBI |
| **hsa-miR-5090** | *LRWD1* | ex./int./s | - | rs3823658 | G>A | NCBI |
| **hsa-miR-5197-3p** | *CTB-57H20.1* | intronic/s | - | rs77549240 | G>T | NCBI |
| **hsa-miR-5589-3p** | *C3P1* | intronic/s | - | rs116796353 | A>G | NCBI |
| **hsa-miR-5692b** | *MX2* | intronic/as | - | rs451887 | T>C | NCBI |
|  | *NDUFV3* |  |  |  |  |  |
| **MOUSE** | | | | | | |
| **mmu-miR-96** | intergenic | / | - | - | A>T | [12] |
| **mmu-miR-599** | *Vps13b* | intronic/as | - | rs37362582 | A>T | NCBI |
| **mmu-miR-654-5p** | intergenic | / | - | rs36943496 | A>G | NCBI |
| **mmu-miR-698** | *Inpp5b* | exonic/s | - | rs27569360 | G>A | NCBI |
| **mmu-miR-717** | *Gpc3* | intronic/s | - | rs30372501 | A>G | [13], NCBI |
| **mmu-miR-741-5p** | intergenic | / | - | rs31707188 | C>T | NCBI |
| **mmu-miR-743a-3p** | intergenic | / | - | rs29048185 | T>C | NCBI |
| **mmu-miR-1948-5p** | *Ttc39c* | intronic/s | - | rs6363056 | A>G | NCBI |
| **mmu-miR-1953** | intergenic | / | - | rs27346553 | T>C | NCBI |
| **mmu-miR-3058-3p** | *Nudt4* | intronic/s | - | rs45674616 | C>T | NCBI |
| **mmu-miR-3080-5p** | intergenic | / | - | rs32341221 | C>T | NCBI |
| **mmu-miR-3104-5p** | *Brsk2* | exonic/s | - | rs38006026 | G>T | NCBI |
| **mmu-miR-5625-3p** | AC105298.2 | exonic/s | - | rs6377091 | A>G | NCBI |
|  | 4930471M23Rik |  |  |  |  |  |
| **CHICKEN** | | | | | | |
| **gga-miR-1568** | intergenic | / | - | rs14511527 | A>G | [40], NCBI |
| **gga-miR-1614*** | intergenic | / | - | rs15172520 | G>A | [40], NCBI |
| **gga-miR-1644** | *Q6IVU9* | intronic/s | - | rs14076349 | C>T | [40], NCBI |
| **gga-miR-1648*** | intergenic | / | - | rs14281065 | T>- | [40], NCBI |
| **gga-miR-1657** | *RAB38* | intronic/s | - | rs14934924 | A>G | [40], NCBI |
| **gga-miR-1658*** | *Q5ZI61* | intronic/s | - | rs16681031 | C>G | [40], NCBI |
|  |  |  |  | rs16681032 | C>T | [40], NCBI |
| **gga-miR-1658** | *Q5ZI61* | intronic/s | - | rs16681033 | ->G | [40], NCBI |
| ***Unvalidated polymorphisms*** | | | | | | |
| **CHIMPANZEE** | | | | | | |
| **ptr-miR-575** | *SCD5* | intronic/s | - | [rs25272907](http://www.ncbi.nlm.nih.gov/sites/entrez?db=snp&cmd=search&term=rs25272907) | A>G | - |
| **HUMAN** | | | | | | |
| **hsa-miR-513a-5p** | intergenic | / | *CD274* | rs35027589 | **->**G | - |
| **hsa-miR-644b** | *DKC1* | intronic/s | - | rs112159031 | G>A | monomorphic (NCBI) |
| **hsa-miR-941-1** | *DNAJC5* | intronic/s | - | rs113283070 | G>C | - |
| **hsa-miR-1324** | intergenic | / | - | rs113859132 | A>G | - |
| **hsa-miR-3118-1** | *AL583842.3* | intronic/s | - | rs2779672 | A>C | - |
|  | *AL583842.2* | intronic/as |  |  |  |  |
| **hsa-miR-3118-2** | *BX571672.1* | intronic/s | - | **rs2779672** | A>C | - |
|  | *BX571672.4* | intronic/s |  |  |  |  |
|  | *BX571672.2* | intronic/as |  |  |  |  |
| **hsa-miR-3118-3** | *BX004987.4* | intronic/s | - | **rs11488501** | C>T | - |
|  |  |  |  | **rs2779672** | A>C | - |
| **hsa-miR-3118-5** | intergenic | / | - | rs4046830 | C>T | - |
| **hsa-miR-3125** | *TRIB2* | intronic/s | - | rs33977954 | ->A | - |
| **hsa-miR-3156-2-3p** | *ANKRD30B* | intronic/s | - | **rs113478966** | T>C | - |
|  |  |  |  | **rs112428304** | C>T | - |
| **hsa-miR-3161** | *PTPRJ* | intronic/s | - | **rs35834266** | ->A | - |
| **hsa-miR-3610** | *RAD21* | exonic/s | - | rs112072631 | T>G | monomorphic (NCBI) |
|  | *RAD21-AS1* | exonic/as |  |  |  |  |
|  | *MIR3610* | exonic/as |  |  |  |  |
| **hsa-miR-3618** | *DGCR8* | exonic/s | - | rs12159555 | C>G | monomorphic (NCBI) |
| **hsa-miR-3622b-5p** | intergenic | / | - | rs13276615 | C>A | monomorphic (NCBI) |
| **hsa-miR-3688-5p** | *RAPGEF2* | intronic/as | - | rs70962655 | ->C | monomorphic (NCBI) |
| **hsa-miR-3689e** | intergenic | / | - | rs72360796 | large | - |
|  |  |  |  |  | deletion |  |
| **hsa-miR-4305** | *COG6* | intronic/as | - | rs4636784 | C>G | - |
| **hsa-miR-4461** | *PCBD2* | intronic/s | - | rs55678614 | G>A | - |
|  | *CTB-36O1.4* | exonic/as |  |  |  |  |
| **hsa-miR-4464** | intergenic | / | - | rs34899210 | ->G | - |
| **hsa-miR-4472-2** | intergenic | / | - | rs57833593 | A>- | monomorphic (NCBI) |
| **hsa-miR-4490** | intergenic | / | - | rs112937431 | A>G | - |
| **hsa-miR-4525** | *RAB40B* | intronic/s | - | rs34711227 | ->C | - |
| **hsa-miR-5007-3p** | intergenic | / | - | rs12865044 | G>C | monomorphic (NCBI) |
| **hsa-miR-5194** | *FAM49B* | intronic/s | - | rs35804210 | C>- | - |
| **MOUSE** | | | | | | |
| **mmu-miR-344h-1-3p** | AC091333.6 | exonic/s | - | rs107861849 | A>T | - |
| **mu-miR-344h-2-3p** | AC091333.6 | exonic/s | - | rs107861849 | A>T | - |
| **mmu-miR-466q** | *WU:Tnik* | intronic/s | - | **rs52447291** | C>T | - |
|  | *Tnik* |  |  | **rs52368901** | A>G | - |
| **RAT** | | | | | | |
| **rno-miR-344b-2** | intergenic | / | - | rs106273682 | C>T | - |
| **ZEBRA FINCH** | | | | | | |
| **tgu-miR-2988** | *VIPR1* | intronic/s | - | [rs83816198](http://www.ncbi.nlm.nih.gov/sites/entrez?db=snp&cmd=search&term=rs83816198) | A>C | - |

*: minor miRNA, ex.: exonic miRNA, int.: intronic miRNA; Host gene orientation: s – sense, as – antisense orientation; /: not applicable; -: data not available; ^1^ miRNA host genes and genomic locations according to the miRBase; ^2^ SNP NCBI ID or as designated in the reference; Consecutive SNPs are shown in bold: 1) miRNAs with both consecutive SNPs validated: hsa-miR-96 (+13 G>A and +14 C>A), hsa-miR-518e-5p (rs34416818 and rs74177814), hsa-miR-1304-5p (rs76857625 and rs79759099), and hsa-miR-3939 (rs75823810 and rs73024232); 2) miRNAs with both consecutive SNPs unvalidated: hsa-miR-3118-3 (rs11488501 and rs2779672), hsa-miR-3159-2-3p (rs113478966 and rs112428304), and mmu-miR-466q (rs52447291 and rs52368901); 3) miRNAs with validated/unvalidated consecutive SNPs: hsa-miR-3118-2 has a validated (rs61786895) and unvalidated (rs2779672) consecutive SNP; hsa-miR-3161 has a validated (rs11382316) and unvalidated (rs35834266) consecutive SNPs.
