# Supplementary material for: Catalog of MicroRNA Seed Polymorphisms in Vertebrates
Source: PLoS One. 2012 Jan 27;7(1):e30737. doi: 10.1371/journal.pone.0030737 (PMC3267754; doi:10.1371/journal.pone.0030737)
Supplement: Table S2 — Estimated differences between miR-seed- SNP (rs30372501) alleles (C>T), associated standard errors and P_values for 363 traits. (DOC) [file pone.0030737.s006.doc]

**Table S2.** Estimated differences between miR-seed- SNP (rs30372501) alleles (C>T), associated standard errors and P_values for 363 traits

| **Measurement number** | **MPD project** | **Variable name** | **Description** | **Units** | **Group** | **Estimate** | **Standard error** | **P_value** |
| --- | --- | --- | --- | --- | --- | --- | --- | --- |
| 1404 | Johnson1 | pip16kHz_yng | ABR threshold for 16 kHz pip stimulus (sound pressure level) | dB | ear | 12.14 | 1.78 | < 0.0001 |
| 1406 | Johnson1 | age_old | age of older group | d | local experiment parameter | -173.83 | 22.34 | < 0.0001 |
| 1408 | Johnson1 | pip8kHz_old | ABR threshold for 8 kHz pip stimulus (sound pressure level) | dB | ear | 25.54 | 3.29 | < 0.0001 |
| 1410 | Johnson1 | pip32kHz_old | ABR threshold for 32 kHz pip stimulus (sound pressure level) | dB | ear | 22.11 | 3.16 | < 0.0001 |
| 1606 | Schulz1 | IC | inspiratory capacity (+ 25 cm H<sub>2</sub>O) | &micro | ungrouped | -99.74 | 5.04 | < 0.0001 |
| 1607 | Schulz1 | FRC | functional residual capacity | &micro | ungrouped | -67.79 | 1.84 | < 0.0001 |
| 1608 | Schulz1 | TLC | total lung capacity | &micro | ungrouped | -165.66 | 6.66 | < 0.0001 |
| 1609 | Schulz1 | VC | vital capacity | &micro | ungrouped | -120.23 | 5.67 | < 0.0001 |
| 1610 | Schulz1 | Cdyn130 | dynamic compliance of respiratory system with breathing rate of 130 per min (H<sub>2</sub>O pressure) | &micro | ungrouped | -4.86 | 0.24 | < 0.0001 |
| 1611 | Schulz1 | CRS | static compliance of respiratory system (H<sub>2</sub>O pressure) | &micro | ungrouped | -9.38 | 0.41 | < 0.0001 |
| 1612 | Schulz1 | CL | static compliance of lung (H<sub>2</sub>O pressure) | &micro | ungrouped | -21.01 | 1.14 | < 0.0001 |
| 1614 | Schulz1 | sR | specific respiratory system resistance with breathing rate of 130 per min related to total lung capacity (H<sub>2</sub>O pressure) | cm&sdot | ungrouped | -0.14 | 0.02 | < 0.0001 |
| 1616 | Schulz1 | slope | intrapulmonary gas mixing: slope of phase III (Hg pressure) | mm/mL | respiratory | -0.07 | 0.01 | < 0.0001 |
| 1617 | Schulz1 | VA | alveolar volume | &micro | ungrouped | 75.83 | 5.66 | < 0.0001 |
| 1618 | Schulz1 | D_CO | diffusing capacity: carbon monoxide | &micro | ungrouped | 1.12 | 0.06 | < 0.0001 |
| 1622 | Schulz1 | ratio_TLC_bw | total lung capacity to body weight | &micro | ungrouped | -5.18 | 0.29 | < 0.0001 |
| 1623 | Schulz1 | ratio_VC_bw | vital capacity to body weight | L/g | respiratory | -3.57 | 0.24 | < 0.0001 |
| 1624 | Schulz1 | ratio_FRC_bw | functional residual capacity to body weight | &micro | ungrouped | -2.28 | 0.08 | < 0.0001 |
| 1630 | Schulz1 | ratio_VDF_TLC | Fowler dead space volume to total lung capacity | &micro | ungrouped | 0.02 | 0 | < 0.0001 |
| 1632 | Schulz1 | VDF | Fowler dead space volume | &micro | ungrouped | -2.99 | 0.45 | < 0.0001 |
| 2905 | Paigen1 | initbw | body weight | g | body weight size and growth | 1.93 | 0.08 | < 0.0001 |
| 2906 | Paigen1 | finalbw | final body weight | g | body weight size and growth | 6.58 | 0.1 | < 0.0001 |
| 2907 | Paigen1 | liverweight | liver weight at sacrifice | g | liver | 0.46 | 0.01 | < 0.0001 |
| 2912 | Paigen1 | smallLC | small liquid crystals (score: 0-4) | score | gallbladder | -0.36 | 0.02 | < 0.0001 |
| 2915 | Paigen1 | ChMC | cholesterol monohydrate crystals (score: 0-4) | score | gallbladder | -0.29 | 0.04 | < 0.0001 |
| 2921 | Paigen1 | HDL_Ch | HDL cholesterol (plasma) | mg/dL | blood--lipids | 20.41 | 0.87 | < 0.0001 |
| 2923 | Paigen1 | pctHDL-TCH | percent of total plasma cholesterol in HDL fraction | % | blood--lipids | 13.73 | 0.39 | < 0.0001 |
| 2924 | Paigen1 | ALT | alanine aminotransferase (plasma ALT) | IU/L | blood--clinical chemistry | -140.51 | 5.03 | < 0.0001 |
| 2926 | Paigen1 | freeHCC | hepatic cholesterol concentration (free per g liver) | mg | liver | -1.5 | 0.05 | < 0.0001 |
| 6205 | Peters1 | MCV | mean RBC corpuscular volume (MCV) | fL | blood--hematology | 0.52 | 0.05 | < 0.0001 |
| 6209 | Peters1 | RDW | RBC corpuscular distribution width (RDW) | % | blood--hematology | -2 | 0.06 | < 0.0001 |
| 6210 | Peters1 | HDW | hemoglobin concentration distribution width (HDW) | g/dL | blood--hematology | -0.48 | 0.03 | < 0.0001 |
| 6213 | Peters1 | MONO | percent monocytes (percent of total number of leukocytes) | % | blood--hematology | -0.16 | 0.01 | < 0.0001 |
| 6219 | Peters1 | PLT | platelet (PLT) count (units per volume x 10<sup>3</sup>) | n/&micro | ungrouped | -197.09 | 6.63 | < 0.0001 |
| 6227 | Peters1 | PTT | partial thromboplastin time (PTT) | s | blood--hematology | 1.38 | 0.07 | < 0.0001 |
| 9101 | Willott1 | bw | body weight | g | body weight size and growth | 4.68 | 0.09 | < 0.0001 |
| 9109 | Willott1 | PPI_12 | mean prepulse inhibition (PPI). 70 dB at 12 kHz | neurosensory | sensory gating | -0.11 | 0.01 | < 0.0001 |
| 9204 | Seburn1 | age | animal age at test | d | local experiment parameter | 2.17 | 0.12 | < 0.0001 |
| 9205 | Seburn1 | bw_in | body weight at start of testing | g | body weight size and growth | 2.81 | 0.1 | < 0.0001 |
| 9206 | Seburn1 | bw_out | body weight at end of testing | g | body weight size and growth | 2.27 | 0.1 | < 0.0001 |
| 9224 | Seburn1 | tot_light | total. light 12h period (beam breaks per minute) | n/min | behavior | 5.85 | 0.38 | < 0.0001 |
| 9419 | Brown1 | urination_LD | urine puddle count | n | behavior | 0.5 | 0.05 | < 0.0001 |
| 9421 | Brown1 | close_dur_EPM | closed arms duration | s | behavior | 23.31 | 1.91 | < 0.0001 |
| 9423 | Brown1 | close_prop_EPM | closed arms. proportion of total time | proportion | behavior | 0.08 | 0.01 | < 0.0001 |
| 9436 | Brown1 | urination_EZM | urine puddle count | n | behavior | 0.74 | 0.07 | < 0.0001 |
| 9455 | Brown1 | open_dur_EPM | open arms duration | s | behavior | -30.02 | 1.99 | < 0.0001 |
| 9458 | Brown1 | open_prop_EPM | open arms. proportion of total time | proportion | behavior | -0.1 | 0.01 | < 0.0001 |
| 9470 | Brown1 | bw_OFT | body weight before open field test | g | local experiment parameter | 4.35 | 0.08 | < 0.0001 |
| 9904 | Paigen2 | HDLC | HDL cholesterol | mg/dL | blood--lipids | 8.62 | 0.39 | < 0.0001 |
| 9906 | Paigen2 | CHOL | total cholesterol | mg/dL | blood--lipids | 29.19 | 0.62 | < 0.0001 |
| 9907 | Paigen2 | HDLC_fat17 | HDL cholesterol | mg/dL | blood--lipids | 35.28 | 1 | < 0.0001 |
| 9909 | Paigen2 | CHOL_fat17 | total cholesterol | mg/dL | blood--lipids | -103.44 | 5.85 | < 0.0001 |
| 9910 | Paigen2 | HDLC_chg | fold change in HDL cholesterol | ratio | blood--lipids | 0.26 | 0.02 | < 0.0001 |
| 9921 | Paigen2 | TG | triglycerides | mg/dL | blood--lipids | 23.8 | 1.17 | < 0.0001 |
| 10305 | Tordoff3 | bw_start | body weight at start of testing | g | body weight size and growth | 5.83 | 0.07 | < 0.0001 |
| 10306 | Tordoff3 | bw_end | body weight at end of testing | g | body weight size and growth | 5.83 | 0.07 | < 0.0001 |
| 10322 | Tordoff3 | pH | blood pH | pH | blood--clinical chemistry | 0.04 | 0 | < 0.0001 |
| 10324 | Tordoff3 | total_calcium | plasma total calcium | mg/dL | blood--clinical chemistry | -0.14 | 0.02 | < 0.0001 |
| 10326 | Tordoff3 | BMD | bone mineral density | g/cm<sup>2</sup> | bone | 0.01 | 0 | < 0.0001 |
| 10327 | Tordoff3 | BMC | bone mineral content | g | bone | 0.1 | 0 | < 0.0001 |
| 10328 | Tordoff3 | lean_wt | calculated weight of lean tissue | g | body composition | 2.93 | 0.05 | < 0.0001 |
| 10329 | Tordoff3 | fat_wt | calculated weight of fat tissue | g | body composition | 2.84 | 0.06 | < 0.0001 |
| 10330 | Tordoff3 | total_wt | total weight (lean + fat) | g | body composition | 5.78 | 0.09 | < 0.0001 |
| 10401 | Deschepper1 | bw | body weight | g | body weight size and growth | 3.37 | 0.08 | < 0.0001 |
| 10402 | Deschepper1 | BP | systolic blood pressure | mmHg | cardiovascular | 4.26 | 0.42 | < 0.0001 |
| 10403 | Deschepper1 | heart_RV | ventricle (right) weight | mg | cardiovascular | 1.51 | 0.15 | < 0.0001 |
| 10405 | Deschepper1 | heart_LV | ventricle (left) weight | mg | cardiovascular | 9.3 | 0.35 | < 0.0001 |
| 10407 | Deschepper1 | heart_biV | ventricles weight | mg | cardiovascular | 10.62 | 0.4 | < 0.0001 |
| 10415 | Deschepper1 | thymus | thymus weight | mg | immune system | 18.44 | 0.73 | < 0.0001 |
| 10416 | Deschepper1 | thymus_adj | thymus weight. per gram of body weight | mg | immune system | 0.49 | 0.04 | < 0.0001 |
| 10418 | Deschepper1 | adrenal_R_adj | adrenal (right) weight. per gram of body weight | mg | endocrine | -0.02 | 0 | < 0.0001 |
| 10422 | Deschepper1 | adrenal_bi_adj | adrenals weight. per gram of body weight | mg | endocrine | -0.03 | 0 | < 0.0001 |
| 10423 | Deschepper1 | kidney_R | kidney (right) weight | mg | kidney | 14.91 | 0.99 | < 0.0001 |
| 10424 | Deschepper1 | kidney_R_adj | kidney (right) weight. per gram of body weight | mg | kidney | -0.71 | 0.04 | < 0.0001 |
| 10425 | Deschepper1 | kidney_L | kidney (left) weight | mg | kidney | 18.3 | 0.98 | < 0.0001 |
| 10426 | Deschepper1 | kidney_L_adj | kidney (left) weight. per gram of body weight | mg | kidney | -0.49 | 0.04 | < 0.0001 |
| 10427 | Deschepper1 | kidney_bi | kidneys weight | mg | kidney | 33.26 | 1.89 | < 0.0001 |
| 10428 | Deschepper1 | kidney_bi_adj | kidneys weight. per gram of body weight | mg | kidney | -1.21 | 0.07 | < 0.0001 |
| 10803 | Wahlsten1 | bw | body weight. end of testing | g | local experiment parameter | 3.77 | 0.07 | < 0.0001 |
| 10850 | Wahlsten1 | close | time spent in two closed arms | s | behavior | -9.29 | 0.43 | < 0.0001 |
| 10851 | Wahlsten1 | defecation_EPM | number fecal boli. 5 min trial | n | behavior | -0.84 | 0.08 | < 0.0001 |
| 10854 | Wahlsten1 | center | time spent in center 5 x 5 cm hub | s | behavior | 5.01 | 0.31 | < 0.0001 |
| 11001 | Everett1 | bw | body weight | g | body weight size and growth | 1.44 | 0.09 | < 0.0001 |
| 11004 | Everett1 | post_length | mandible. menton to gonion | mm | bone | 0.08 | 0.01 | < 0.0001 |
| 11005 | Everett1 | mid_length | mandible. infradentale to posterior ramus | mm | bone | 0.27 | 0.01 | < 0.0001 |
| 11006 | Everett1 | mid_height | mandible. posterior molar to menton | mm | bone | 0.12 | 0 | < 0.0001 |
| 11007 | Everett1 | condyle | mandible. condylar process | mm | bone | 0.1 | 0 | < 0.0001 |
| 11009 | Everett1 | incisor_gonion | mandible. infradentale to gonion | mm | bone | 0.33 | 0.01 | < 0.0001 |
| 11012 | Everett1 | incisor_region | mandible. diastema to pogonion | mm | bone | -0.02 | 0 | < 0.0001 |
| 11014 | Everett1 | ant_area | mandible. anterior area | mm<sup>2</sup> | bone | 1 | 0.03 | < 0.0001 |
| 11016 | Everett1 | teeth_space | mandible. concavity of incisor to anterior molar | mm<sup>2</sup> | bone | 0.13 | 0 | < 0.0001 |
| 11017 | Everett1 | ramus_space | mandible. concavity of posterior ramus | mm<sup>2</sup> | bone | 0.25 | 0.01 | < 0.0001 |
| 11506 | Donahue1 | body_BMD | whole body bone mineral density | g/cm<sup>2</sup> | bone | 0.01 | 0 | < 0.0001 |
| 11508 | Donahue1 | body_BMC | whole body bone mineral content | g | bone | 0.12 | 0 | < 0.0001 |
| 11510 | Donahue1 | lean_wt | calculated lean body tissue mass | g | body composition | 3.62 | 0.05 | < 0.0001 |
| 11512 | Donahue1 | fat_wt | weight of fat portion of body mass | g | body composition | 1.22 | 0.04 | < 0.0001 |
| 11514 | Donahue1 | total_wt | calculated total tissue mass | g | body composition | 4.84 | 0.08 | < 0.0001 |
| 11516 | Donahue1 | spine_BMD | spine (L2-L5) bone mineral density | g/cm<sup>2</sup> | bone | 0.01 | 0 | < 0.0001 |
| 11517 | Donahue1 | skull_BMD | skull bone mineral density | g/cm<sup>2</sup> | bone | 0.01 | 0 | < 0.0001 |
| 11518 | Donahue1 | skull_length | total skull length | mm | bone | 1.14 | 0.01 | < 0.0001 |
| 11519 | Donahue1 | nose_length | nose length | mm | bone | 0.56 | 0.01 | < 0.0001 |
| 11520 | Donahue1 | skull_height | skull height | mm | bone | 0.65 | 0.01 | < 0.0001 |
| 11521 | Donahue1 | skull_width | skull width | mm | bone | 0.62 | 0.01 | < 0.0001 |
| 11523 | Donahue1 | upper_jaw | length of upper jaw | mm | bone | 0.61 | 0.01 | < 0.0001 |
| 11524 | Donahue1 | lower_jaw | length of lower jaw | mm | bone | 0.48 | 0.01 | < 0.0001 |
| 11530 | Donahue1 | osteocalcin | osteocalcin (serum OC) | ng/mL | endocrine | -5.91 | 0.62 | < 0.0001 |
| 11618 | Graubert1 | lymphoid_tumor | lymphoid tumor frequency rate (score 0-1) | score | cancer | 0.06 | 0.01 | < 0.0001 |
| 11807 | Flaherty1 | beam_breaks | number beam breaks in closed quadrants | n/s | behavior | 55.43 | 4.11 | < 0.0001 |
| 13204 | Justice2 | WBC | white blood cell count (WBC) (units per volume x 10<sup>3</sup>) | n/&micro | ungrouped | 0.66 | 0.07 | < 0.0001 |
| 13209 | Justice2 | MCH | mean RBC corpuscular hemoglobin (MCH) | pg | blood--hematology | 0.1 | 0.01 | < 0.0001 |
| 13210 | Justice2 | MCHC | mean RBC corpuscular hemoglobin concentration (MCHC) | g/dL | blood--hematology | -0.29 | 0.02 | < 0.0001 |
| 13211 | Justice2 | PLT | platelet (PLT) count (units per volume x 10<sup>3</sup>) | n/&micro | ungrouped | -92.69 | 5.7 | < 0.0001 |
| 13214 | Justice2 | pctN | percent neutrophils (percent of total number of leukocytes) | % | blood--hematology | -1.65 | 0.17 | < 0.0001 |
| 13215 | Justice2 | pctL | percent lymphocytes (percent of total number of leukocytes) | % | blood--hematology | 2.16 | 0.19 | < 0.0001 |
| 13217 | Justice2 | pctE | percent eosinophils (percent of total number of leukocytes) | % | blood--hematology | 0.7 | 0.07 | < 0.0001 |
| 14302 | Naggert1 | GLU | glucose (plasma. 4h fast) | mg/dL | blood--clinical chemistry | 19.35 | 1.22 | < 0.0001 |
| 14303 | Naggert1 | GLU_fat17 | glucose (plasma. 4h fast) | mg/dL | blood--clinical chemistry | 31.5 | 1.9 | < 0.0001 |
| 14305 | Naggert1 | INS_fat18 | insulin | ng/mL | endocrine | 0.77 | 0.03 | < 0.0001 |
| 14308 | Naggert1 | bw_fat8 | body weight | g | body weight size and growth | 7.15 | 0.09 | < 0.0001 |
| 14312 | Naggert1 | BMD_fat8 | bone mineral density (BMD) | g/cm<sup>2</sup> | bone | 0.01 | 0 | < 0.0001 |
| 14314 | Naggert1 | BMC_fat8 | bone mineral content (BMC) | g | bone | 0.11 | 0 | < 0.0001 |
| 14316 | Naggert1 | tissuemass_fat8 | total tissue mass | g | body composition | 7.28 | 0.1 | < 0.0001 |
| 14317 | Naggert1 | fatwt_fat8 | weight of fat portion of tissue mass | g | body composition | 3.8 | 0.07 | < 0.0001 |
| 14318 | Naggert1 | leanwt_fat8 | weight of lean portion of tissue mass | g | body composition | 3.48 | 0.05 | < 0.0001 |
| 15102 | Jaxwest1 | QRS | interval between beginning and end of QRS complex | ms | cardiovascular | -1.53 | 0.16 | < 0.0001 |
| 15121 | Jaxwest1 | tension_mean | grip strength mean peak tension over 5 trials | g | muscle | 28.74 | 1.51 | < 0.0001 |
| 15122 | Jaxwest1 | tension_max | grip strength maximum peak tension over 5 trials | g | muscle | 32.16 | 1.59 | < 0.0001 |
| 15127 | Jaxwest1 | fat_wt | calculated fat weight | g | body composition | -1.2 | 0.07 | < 0.0001 |
| 15130 | Jaxwest1 | BMD | bone mineral density (BMD) | g/cm<sup>2</sup> | bone | 0.01 | 0 | < 0.0001 |
| 15131 | Jaxwest1 | BMC | bone mineral content (BMC) | g | bone | 0.12 | 0 | < 0.0001 |
| 15133 | Jaxwest1 | BMC_femurR | bone mineral content (BMC) of right femur | g | bone | 0 | 0 | < 0.0001 |
| 15409 | Crabbe1 | bal12_doseB | missteps crossing 12.7 mm balance beam | n | behavior | 1.79 | 0.25 | < 0.0001 |
| 17103 | Churchill1 | spine_BMD | vetebral spine bone mineral density | g/cm<sup>2</sup> | bone | 0.02 | 0 | < 0.0001 |
| 17109 | Churchill1 | quadricep_wt | weight of isolated quadriceps muscle | g | muscle | -0.02 | 0 | < 0.0001 |
| 17201 | Donahue2 | bw | whole body weight (with head) | g | body weight size and growth | 1.16 | 0.16 | < 0.0001 |
| 17203 | Donahue2 | femur_cortical_BMD | cortical shell volumetric bone mineral density (vBMD). whole left femur | g/cm<sup>3</sup> | bone | 0.03 | 0 | < 0.0001 |
| 17206 | Donahue2 | femur_peak_load | maximum load on load-displacement curve | N | bone | -2.48 | 0.22 | < 0.0001 |
| 17208 | Donahue2 | femur_break_load | load at specimen failure (break point) | N | bone | -1.87 | 0.28 | < 0.0001 |
| 17213 | Donahue2 | femur_length | length of right femur (condyles to rostral tip of greater trochanter) | mm | bone | 0.39 | 0.02 | < 0.0001 |
| 17214 | Donahue2 | femur_width_AP | anterior to posterior (rostral to caudal) width of right femur (mid-diaphysis) | mm | bone | 0.04 | 0.01 | < 0.0001 |
| 17216 | Donahue2 | femur_area | cross sectional bone area (whole thigh area minus muscle area). femoral mid-diaphysis | mm<sup>2</sup> | bone | 0.09 | 0.01 | < 0.0001 |
| 17217 | Donahue2 | femur_thickness_R1 | cortical thickness of right femur. pQCT | mm | bone | -0.02 | 0 | < 0.0001 |
| 17218 | Donahue2 | femur_thickness_R2 | cortical thickness of right femur. &micro | -0.03 | 0 | -0.03 | 0 | < 0.0001 |
| 17220 | Donahue2 | femur_out | periosteal (outer) circumference. femoral mid-diaphysis | mm | bone | 0.12 | 0.01 | < 0.0001 |
| 17221 | Donahue2 | femur_in | endosteal (inner) circumference. femoral mid-diaphysis | mm | bone | 0.25 | 0.01 | < 0.0001 |
| 17222 | Donahue2 | thigh_area | cross-sectional area of thigh muscle and bone. femoral mid-diaphysis | mm<sup>2</sup> | muscle | 5.52 | 0.68 | < 0.0001 |
| 17223 | Donahue2 | thigh_muscle_area | cross-sectional thigh muscle area (thigh area minus femur area). femoral mid-diaphysis | mm<sup>2</sup> | muscle | 5.43 | 0.67 | < 0.0001 |
| 17224 | Donahue2 | quadricep_wt | weight of isolated right quadriceps muscle | g | muscle | 0.02 | 0 | < 0.0001 |
| 17230 | Donahue2 | elastic_modulus | estimated elastic modulus | N/mm<sup>2</sup> | bone | -0.59 | 0.05 | < 0.0001 |
| 17231 | Donahue2 | ultimate_strength | estimated ultimate strength | N | bone | -37.5 | 2.88 | < 0.0001 |
| 17704 | Svenson1 | bp_sd | systolic blood pressure variability across tests | mmHg | local experiment parameter | -2.77 | 0.41 | < 0.0001 |
| 19106 | Crabbe4 | gripbaseline | peak force applied. baseline grip strength test | g | muscle | 12.25 | 0.89 | < 0.0001 |
| 19113 | Crabbe4 | BEC_grip60 | blood ethanol concentration (BEC) 60 min post-injection. grip strength test | mg/mL | blood--miscellaneous | 0.12 | 0.01 | < 0.0001 |
| 19701 | Paigen4 | HDLC | HDL cholesterol | mg/dL | blood--lipids | 30.22 | 0.38 | < 0.0001 |
| 19702 | Paigen4 | CHOL | total cholesterol | mg/dL | blood--lipids | 29.44 | 0.53 | < 0.0001 |
| 19704 | Paigen4 | TG | triglycerides | mg/dL | blood--lipids | 16.03 | 0.92 | < 0.0001 |
| 21402 | Wiltshire1 | rear_OFT | total rearing events. 10 min | n | behavior | 21.33 | 0.8 | < 0.0001 |
| 22401 | Jaxpheno1 | bw8 | body weight | g | body weight size and growth | 2.1 | 0.13 | < 0.0001 |
| 22410 | Jaxpheno1 | tissue_mass8 | total body tissue mass (calculated) | g | body composition | 2.01 | 0.14 | < 0.0001 |
| 22412 | Jaxpheno1 | lean_wt8 | lean body tissue mass (derived) | g | body composition | 2.19 | 0.1 | < 0.0001 |
| 22413 | Jaxpheno1 | lean_wt16 | lean body tissue mass (derived) | g | body composition | 0.93 | 0.1 | < 0.0001 |
| 22415 | Jaxpheno1 | fat_wt16 | body fat tissue weight (calculated) | g | body composition | -1.23 | 0.1 | < 0.0001 |
| 22430 | Jaxpheno1 | body_BMC8 | whole body bone mineral content (without head) | g | bone | 0.07 | 0 | < 0.0001 |
| 22431 | Jaxpheno1 | body_BMC16 | whole body bone mineral content (without head) | g | bone | 0.05 | 0 | < 0.0001 |
| 22440 | Jaxpheno1 | bone_area8 | whole body bone area (without head) | cm<sup>2</sup> | bone | 0.98 | 0.03 | < 0.0001 |
| 22441 | Jaxpheno1 | bone_area16 | whole body bone area (without head) | cm<sup>2</sup> | bone | 1.15 | 0.04 | < 0.0001 |
| 22512 | Brown2 | acq_err_d4 | acquisition training mean errors. day 4 | n | behavior | 4.25 | 0.28 | < 0.0001 |
| 22515 | Brown2 | acq_lat_d1 | acquisition training mean latency to escape. day 1 | s | behavior | 44.94 | 2.51 | < 0.0001 |
| 22561 | Brown2 | fall_ave_d1 | latency to fall from accelerating (8rpm/min) rotarod. day 1 | s | behavior | -10.12 | 1.26 | < 0.0001 |
| 22563 | Brown2 | fall_ave_d3 | latency to fall from accelerating (8rpm/min) rotarod. day 3 | s | behavior | 20.28 | 1.76 | < 0.0001 |
| 22564 | Brown2 | fall_ave_d4 | latency to fall from accelerating (8rpm/min) rotarod. day 4 | s | behavior | 25.54 | 1.83 | < 0.0001 |
| 22565 | Brown2 | fall_ave_d5 | latency to fall from accelerating (8rpm/min) rotarod. day 5 | s | behavior | 18.49 | 2.02 | < 0.0001 |
| 22566 | Brown2 | fall_ave_d6 | latency to fall from accelerating (8rpm/min) rotarod. day 6 | s | behavior | 13.89 | 1.99 | < 0.0001 |
| 22567 | Brown2 | fall_ave_d7 | latency to fall from accelerating (8rpm/min) rotarod. day 7 | s | behavior | 13.9 | 2.04 | < 0.0001 |
| 22703 | Jaxpheno2 | pct_brain8 | brain weight as percent of body weight | % | brain | 0.11 | 0.01 | < 0.0001 |
| 22704 | Jaxpheno2 | pct_brain16 | brain weight as percent of body weight | % | brain | 0.23 | 0.01 | < 0.0001 |
| 22721 | Jaxpheno2 | liver16 | liver weight | g | liver | 0.14 | 0.02 | < 0.0001 |
| 22730 | Jaxpheno2 | kidney_L8 | kidney (left) weight | g | kidney | 0.02 | 0 | < 0.0001 |
| 22731 | Jaxpheno2 | kidney_L16 | kidney (left) weight | g | kidney | 0.02 | 0 | < 0.0001 |
| 22735 | Jaxpheno2 | pct_kidney_L16 | kidney (left) weight as percent of body weight | % | kidney | 0.07 | 0.01 | < 0.0001 |
| 22741 | Jaxpheno2 | spleen16 | spleen weight | g | spleen | 0.01 | 0 | < 0.0001 |
| 22743 | Jaxpheno2 | pct_spleen16 | spleen weight as percent of body weight | % | spleen | 0.02 | 0 | < 0.0001 |
| 22750 | Jaxpheno2 | bw8 | body weight | g | body weight size and growth | 1.6 | 0.12 | < 0.0001 |
| 22801 | Jaxpheno3 | GLU8 | glucose (plasma GLU. 4h fast) | mg/dL | blood--clinical chemistry | -15.13 | 1.33 | < 0.0001 |
| 22810 | Jaxpheno3 | ALB8 | albumin (plasma Alb) | g/dL | blood--clinical chemistry | -0.37 | 0.02 | < 0.0001 |
| 22813 | Jaxpheno3 | TP16 | total protein (plasma TP) | g/dL | blood--clinical chemistry | -0.3 | 0.03 | < 0.0001 |
| 22830 | Jaxpheno3 | CHOL8 | total cholesterol (plasma CHOL) | mg/dL | blood--lipids | 18.99 | 0.48 | < 0.0001 |
| 22831 | Jaxpheno3 | CHOL16 | total cholesterol (plasma CHOL) | mg/dL | blood--lipids | 26.89 | 1.91 | < 0.0001 |
| 22835 | Jaxpheno3 | HDL8 | high density lipoprotein cholesterol (plasma HDL) direct | mg/dL | blood--lipids | 14.53 | 0.42 | < 0.0001 |
| 22836 | Jaxpheno3 | HDL16 | high density lipoprotein cholesterol (plasma HDL) direct | mg/dL | blood--lipids | 11.08 | 0.98 | < 0.0001 |
| 22851 | Jaxpheno3 | Ca16 | calcium (plasma Ca) | mg/dL | blood--clinical chemistry | -0.28 | 0.03 | < 0.0001 |
| 22901 | Jaxpheno4 | WBC8 | total white blood cell (WBC. leukocyte) count (units per volume x 10<sup>3</sup>) | n/&micro | ungrouped | -0.67 | 0.05 | < 0.0001 |
| 22903 | Jaxpheno4 | RBC8 | total red blood cell (RBC. erythrocyte) count (units per volume x 10<sup>6</sup>) | n/&micro | ungrouped | -0.55 | 0.02 | < 0.0001 |
| 22904 | Jaxpheno4 | RBC16 | total red blood cell (RBC. erythrocyte) count (units per volume x 10<sup>6</sup>) | n/&micro | ungrouped | -0.63 | 0.02 | < 0.0001 |
| 22912 | Jaxpheno4 | LYMPH8 | lymphocyte (LYMP) count (units per volume x 10<sup>3</sup>) | n/&micro | ungrouped | -0.48 | 0.04 | < 0.0001 |
| 22913 | Jaxpheno4 | LYMPH16 | lymphocyte (LYMP) count (units per volume x 10<sup>3</sup>) | n/&micro | ungrouped | -0.85 | 0.04 | < 0.0001 |
| 22935 | Jaxpheno4 | pct_MONO16 | percent monocytes (percent of total WBC) | % | blood--hematology | 0.32 | 0.05 | < 0.0001 |
| 22950 | Jaxpheno4 | PLT8 | platelet (PLT) count (units per volume x 10<sup>3</sup>) | n/&micro | ungrouped | 196.21 | 8.74 | < 0.0001 |
| 22951 | Jaxpheno4 | PLT16 | platelet (PLT) count (units per volume x 10<sup>3</sup>) | n/&micro | ungrouped | 196.93 | 9.33 | < 0.0001 |
| 22952 | Jaxpheno4 | MPV8 | mean platelet volume (MPV) | fL | blood--hematology | -0.65 | 0.03 | < 0.0001 |
| 22969 | Jaxpheno4 | HCT16 | hematocrit (HCT) | % | blood--hematology | 0.78 | 0.13 | < 0.0001 |
| 22970 | Jaxpheno4 | MCV8 | mean RBC corpuscular volume (MCV) | fL | blood--hematology | 2.58 | 0.05 | < 0.0001 |
| 22971 | Jaxpheno4 | MCV16 | mean RBC corpuscular volume (MCV) | fL | blood--hematology | 3.54 | 0.06 | < 0.0001 |
| 22977 | Jaxpheno4 | CHr8 | reticulocyte corpuscular hemoglobin content (CHr) | pg | blood--hematology | 1.14 | 0.02 | < 0.0001 |
| 22978 | Jaxpheno4 | CHr16 | reticulocyte corpuscular hemoglobin content (CHr) | pg | blood--hematology | 1.24 | 0.02 | < 0.0001 |
| 22982 | Jaxpheno4 | MCH8 | mean RBC corpuscular hemoglobin content (MCH) | pg | blood--hematology | 0.75 | 0.03 | < 0.0001 |
| 22983 | Jaxpheno4 | MCH16 | mean RBC corpuscular hemoglobin content (MCH) | pg | blood--hematology | 0.6 | 0.03 | < 0.0001 |
| 22985 | Jaxpheno4 | MCHC16 | mean RBC corpuscular hemoglobin concentration (MCHC) | g/dL | blood--hematology | -1.23 | 0.06 | < 0.0001 |
| 23002 | Jaxpheno5 | bw04 | body weight | g | body weight size and growth | 4.23 | 0.06 | < 0.0001 |
| 23003 | Jaxpheno5 | bw05 | body weight | g | body weight size and growth | 3.04 | 0.06 | < 0.0001 |
| 23004 | Jaxpheno5 | bw06 | body weight | g | body weight size and growth | 2.35 | 0.06 | < 0.0001 |
| 23005 | Jaxpheno5 | bw07 | body weight | g | body weight size and growth | 2.09 | 0.06 | < 0.0001 |
| 23006 | Jaxpheno5 | bw08 | body weight | g | body weight size and growth | 1.66 | 0.06 | < 0.0001 |
| 23007 | Jaxpheno5 | bw09 | body weight | g | body weight size and growth | 1.37 | 0.06 | < 0.0001 |
| 23008 | Jaxpheno5 | bw10 | body weight | g | body weight size and growth | 1.65 | 0.07 | < 0.0001 |
| 23009 | Jaxpheno5 | bw11 | body weight | g | body weight size and growth | 1.78 | 0.07 | < 0.0001 |
| 23010 | Jaxpheno5 | bw12 | body weight | g | body weight size and growth | 1.48 | 0.07 | < 0.0001 |
| 23011 | Jaxpheno5 | bw13 | body weight | g | body weight size and growth | 1.42 | 0.07 | < 0.0001 |
| 23012 | Jaxpheno5 | bw14 | body weight | g | body weight size and growth | 1.27 | 0.08 | < 0.0001 |
| 23013 | Jaxpheno5 | bw15 | body weight | g | body weight size and growth | 0.76 | 0.09 | < 0.0001 |
| 23103 | Jaxpheno6 | pct_CD8_8 | CD8 T cells (percent of total viable cells) | % | immune system | 1.55 | 0.08 | < 0.0001 |
| 23123 | Jaxpheno6 | pct_Mono16 | monocytes (percent of total viable cells) | % | immune system | 0.36 | 0.04 | < 0.0001 |
| 23130 | Jaxpheno6 | bw8 | body weight | g | body weight size and growth | 1.35 | 0.13 | < 0.0001 |
| 24201 | Petkova1 | WBC_M06 | total white blood cell (WBC. leukocyte) count (units per volume x 10<sup>3</sup>) | n/&micro | ungrouped | -4.15 | 0.22 | < 0.0001 |
| 24206 | Petkova1 | LYM_M06 | lymphocytes (percent of viable WBC) | % | blood--hematology | -7.43 | 0.31 | < 0.0001 |
| 24208 | Petkova1 | LYM_M18 | lymphocytes (percent of viable WBC) | % | blood--hematology | -9.85 | 0.58 | < 0.0001 |
| 24211 | Petkova1 | NEUT_M06 | neutrophils (percent of viable WBC) | % | blood--hematology | 7.63 | 0.33 | < 0.0001 |
| 24212 | Petkova1 | NEUT_M12 | neutrophils (percent of viable WBC) | % | blood--hematology | 5 | 0.51 | < 0.0001 |
| 24213 | Petkova1 | NEUT_M18 | neutrophils (percent of viable WBC) | % | blood--hematology | 9.53 | 0.51 | < 0.0001 |
| 24216 | Petkova1 | EOS_M06 | eosinophils (percent of viable WBC) | % | blood--hematology | 1.12 | 0.07 | < 0.0001 |
| 24226 | Petkova1 | B_cells_M12 | B cells (as percent of total lymphocytes) | % | immune system | -5.29 | 0.44 | < 0.0001 |
| 24233 | Petkova1 | CD4_naive_M06 | naive CD4 T cells (as percent of total CD4 T cells) | % | immune system | -4.61 | 0.51 | < 0.0001 |
| 24238 | Petkova1 | CD4_mem_central_M12 | central memory CD4 T cells (as percent of total CD4 T cells) | % | immune system | 0.68 | 0.07 | < 0.0001 |
| 24247 | Petkova1 | CD4_effector_M12 | effector CD4 T cells (as percent of total CD4 T cells) | % | immune system | -8.52 | 0.56 | < 0.0001 |
| 24251 | Petkova1 | CD8_all_M06 | CD8 T cells (as percent of total lymphocytes) | % | immune system | 1.82 | 0.15 | < 0.0001 |
| 24253 | Petkova1 | CD8_all_M18 | CD8 T cells (as percent of total lymphocytes) | % | immune system | 4.96 | 0.28 | < 0.0001 |
| 24256 | Petkova1 | CD8_naive_M06 | naive CD8 T cells (as percent of total CD8 T cells) | % | immune system | -5.28 | 0.56 | < 0.0001 |
| 24258 | Petkova1 | CD8_naive_M18 | naive CD8 T cells (as percent of total CD8 T cells) | % | immune system | 12.54 | 0.68 | < 0.0001 |
| 24261 | Petkova1 | CD8_mem_central_M06 | central memory CD8 T cells (as percent of total CD8 T cells) | % | immune system | 7.4 | 0.19 | < 0.0001 |
| 24266 | Petkova1 | CD8_mem_effector_M06 | effector memory CD8 T cells (as percent of total CD8 T cells) | % | immune system | -3.84 | 0.29 | < 0.0001 |
| 24267 | Petkova1 | CD8_mem_effector_M12 | effector memory CD8 T cells (as percent of total CD8 T cells) | % | immune system | -3.48 | 0.39 | < 0.0001 |
| 24268 | Petkova1 | CD8_mem_effector_M18 | effector memory CD8 T cells (as percent of total CD8 T cells) | % | immune system | -6.41 | 0.38 | < 0.0001 |
| 24269 | Petkova1 | CD8_mem_effector_M24 | effector memory CD8 T cells (as percent of total CD8 T cells) | % | immune system | -7.79 | 1.07 | < 0.0001 |
| 24276 | Petkova1 | NK_M06 | natural killer (NK) cells (as percent of total lymphocytes) | % | immune system | 1.5 | 0.08 | < 0.0001 |
| 24302 | Peters4 | WBC_M12 | total white blood cell (WBC. leukocyte) count (units per volume x 10<sup>3</sup>) | n/&micro | ungrouped | -0.94 | 0.1 | < 0.0001 |
| 24308 | Peters4 | RBC_M24 | total red blood cell (RBC. erythrocyte) count (units per volume x 10<sup>6</sup>) | n/&micro | ungrouped | 1.48 | 0.09 | < 0.0001 |
| 24310 | Peters4 | NEUT_M12 | neutrophil (NEUT) count (units per volume x 10<sup>3</sup>) | n/&micro | ungrouped | 0.5 | 0.06 | < 0.0001 |
| 24311 | Peters4 | NEUT_M18 | neutrophil (NEUT) count (units per volume x 10<sup>3</sup>) | n/&micro | ungrouped | 1.29 | 0.08 | < 0.0001 |
| 24313 | Peters4 | LYMPH_M06 | lymphocyte (LYMP) count (units per volume x 10<sup>3</sup>) | n/&micro | ungrouped | -1.11 | 0.08 | < 0.0001 |
| 24318 | Peters4 | MONO_M12 | monocyte (MONO) count (units per volume x 10<sup>3</sup>) | n/&micro | ungrouped | -0.08 | 0 | < 0.0001 |
| 24319 | Peters4 | MONO_M18 | monocyte (MONO) count (units per volume x 10<sup>3</sup>) | n/&micro | ungrouped | -0.06 | 0.01 | < 0.0001 |
| 24322 | Peters4 | EOS_M12 | eosinophil (EOS) count (units per volume x 10<sup>3</sup>) | n/&micro | ungrouped | 0.06 | 0.01 | < 0.0001 |
| 24330 | Peters4 | LUC_M12 | large unstained cells (LUC) count (units per volume x 10<sup>3</sup>) | n/&micro | ungrouped | -0.02 | 0 | < 0.0001 |
| 24334 | Peters4 | pctNEUT_M12 | percent neutrophils (percent of total WBC) | % | blood--hematology | 5.78 | 0.4 | < 0.0001 |
| 24335 | Peters4 | pctNEUT_M18 | percent neutrophils (percent of total WBC) | % | blood--hematology | 12.04 | 0.56 | < 0.0001 |
| 24338 | Peters4 | pctLYMPH_M12 | percent lymphocytes (percent of total WBC) | % | blood--hematology | -5.77 | 0.4 | < 0.0001 |
| 24339 | Peters4 | pctLYMPH_M18 | percent lymphocytes (percent of total WBC) | % | blood--hematology | -10.85 | 0.56 | < 0.0001 |
| 24350 | Peters4 | pctBASO_M12 | percent basophils (percent of total WBC) | % | blood--hematology | -0.05 | 0.01 | < 0.0001 |
| 24351 | Peters4 | pctBASO_M18 | percent basophils (percent of total WBC) | % | blood--hematology | -0.1 | 0.01 | < 0.0001 |
| 24358 | Peters4 | pctHCT_M12 | hematocrit (HCT) | % | blood--hematology | 1.78 | 0.12 | < 0.0001 |
| 24359 | Peters4 | pctHCT_M18 | hematocrit (HCT) | % | blood--hematology | 1.09 | 0.14 | < 0.0001 |
| 24371 | Peters4 | MCHC_M18 | mean RBC corpuscular hemoglobin concentration (MCHC) | g/dL | blood--hematology | -0.74 | 0.03 | < 0.0001 |
| 24375 | Peters4 | MCV_M18 | mean RBC corpuscular volume (MCV) | fL | blood--hematology | 1.25 | 0.07 | < 0.0001 |
| 24385 | Peters4 | CHr_M06 | reticulocyte corpuscular hemoglobin content (CHr) | pg | blood--hematology | -0.44 | 0.02 | < 0.0001 |
| 24386 | Peters4 | CHr_M12 | reticulocyte corpuscular hemoglobin content (CHr) | pg | blood--hematology | 0.22 | 0.02 | < 0.0001 |
| 24387 | Peters4 | CHr_M18 | reticulocyte corpuscular hemoglobin content (CHr) | pg | blood--hematology | 0.36 | 0.03 | < 0.0001 |
| 24389 | Peters4 | MPV_M06 | mean platelet volume (MPV) | fL | blood--hematology | 1.09 | 0.03 | < 0.0001 |
| 24394 | Peters4 | Plt_M12 | platelet (PLT) count (units per volume x 10<sup>3</sup>) | n/&micro | ungrouped | -176.3 | 12.34 | < 0.0001 |
| 24395 | Peters4 | Plt_M18 | platelet (PLT) count (units per volume x 10<sup>3</sup>) | n/&micro | ungrouped | -225.17 | 19.96 | < 0.0001 |
| 24402 | Yuan3 | ALT_M12 | alanine aminotransferase (plasma ALT) | IU/L | blood--clinical chemistry | -5.17 | 0.65 | < 0.0001 |
| 24411 | Yuan3 | ALP_M06 | alkaline phosphatase (serum ALP) | IU/L | blood--clinical chemistry | 33.28 | 1.01 | < 0.0001 |
| 24412 | Yuan3 | ALP_M12 | alkaline phosphatase (serum ALP) | IU/L | blood--clinical chemistry | 30.4 | 3.75 | < 0.0001 |
| 24413 | Yuan3 | ALP_M18 | alkaline phosphatase (serum ALP) | IU/L | blood--clinical chemistry | 71.56 | 5.19 | < 0.0001 |
| 24426 | Yuan3 | Fe_M06 | iron (serum Fe) | mmol/L | blood--clinical chemistry | 60.71 | 1.87 | < 0.0001 |
| 24427 | Yuan3 | Fe_M12 | iron (serum Fe) | mmol/L | blood--clinical chemistry | 58.5 | 2.08 | < 0.0001 |
| 24431 | Yuan3 | Mg_M06 | magnesium (serum Mg) | mmol/L | blood--clinical chemistry | 0.21 | 0.01 | < 0.0001 |
| 24441 | Yuan3 | K_M06 | potassium (serum K) | mmol/L | blood--clinical chemistry | -0.34 | 0.04 | < 0.0001 |
| 24446 | Yuan3 | Na_M06 | sodium (serum Na) | mmol/L | blood--clinical chemistry | -2.19 | 0.2 | < 0.0001 |
| 24452 | Yuan3 | ALB_M12 | albumin (plasma Alb) | g/dL | blood--clinical chemistry | -0.22 | 0.01 | < 0.0001 |
| 24456 | Yuan3 | TP_M06 | total protein (plasma TP) | g/dL | blood--clinical chemistry | 0.24 | 0.01 | < 0.0001 |
| 24458 | Yuan3 | TP_M18 | total protein (plasma TP) | g/dL | blood--clinical chemistry | 0.33 | 0.02 | < 0.0001 |
| 24466 | Yuan3 | TBIL_M06 | total bilirubin (serum TBIL) | &micro | ungrouped | 0.07 | 0.01 | < 0.0001 |
| 24467 | Yuan3 | TBIL_M12 | total bilirubin (serum TBIL) | &micro | ungrouped | 0.05 | 0.01 | < 0.0001 |
| 24471 | Yuan3 | HDL_M06 | high density lipoprotein cholesterol (plasma HDL) | mg/dL | blood--lipids | 32.68 | 0.53 | < 0.0001 |
| 24472 | Yuan3 | HDL_M12 | high density lipoprotein cholesterol (plasma HDL) | mg/dL | blood--lipids | 11.03 | 0.67 | < 0.0001 |
| 24473 | Yuan3 | HDL_M18 | high density lipoprotein cholesterol (plasma HDL) | mg/dL | blood--lipids | 39.82 | 1.03 | < 0.0001 |
| 24481 | Yuan3 | BUN_M06 | blood urea nitrogen (plasma BUN) | mg/dL | blood--clinical chemistry | -1.63 | 0.17 | < 0.0001 |
| 24483 | Yuan3 | BUN_M18 | blood urea nitrogen (plasma BUN) | mg/dL | blood--clinical chemistry | -2.35 | 0.26 | < 0.0001 |
| 24802 | Seburn2 | grip_strength_M12 | grip strength mean peak tension over 3 trials | g | muscle | -8.63 | 0.54 | < 0.0001 |
| 24803 | Seburn2 | grip_strength_M18 | grip strength mean peak tension over 3 trials | g | muscle | -6.28 | 0.57 | < 0.0001 |
| 25002 | Ackert1 | BL_M12 | body length (tip of nose to base of tail) | cm | body weight size and growth | 0.22 | 0.01 | < 0.0001 |
| 25006 | Ackert1 | BW_M06 | body weight | g | body weight size and growth | 5.28 | 0.13 | < 0.0001 |
| 25008 | Ackert1 | BW_M20 | body weight | g | body weight size and growth | 2.02 | 0.23 | < 0.0001 |
| 25011 | Ackert1 | BMD_M06 | whole body bone mineral density | g/cm<sup>2</sup> | bone | 0.01 | 0 | < 0.0001 |
| 25016 | Ackert1 | BMC_M06 | whole body bone mineral content | g | bone | 0.08 | 0 | < 0.0001 |
| 25017 | Ackert1 | BMC_M12 | whole body bone mineral content | g | bone | 0.09 | 0 | < 0.0001 |
| 25021 | Ackert1 | bone_area_M06 | whole body bone area (head excluded) | cm<sup>2</sup> | bone | 0.46 | 0.04 | < 0.0001 |
| 25022 | Ackert1 | bone_area_M12 | whole body bone area (head excluded) | cm<sup>2</sup> | bone | 0.71 | 0.04 | < 0.0001 |
| 25026 | Ackert1 | total_area_M06 | total body area (head excluded) | cm<sup>2</sup> | body weight size and growth | 2.38 | 0.08 | < 0.0001 |
| 25027 | Ackert1 | total_area_M12 | total body area (head excluded) | cm<sup>2</sup> | body weight size and growth | 0.85 | 0.1 | < 0.0001 |
| 25031 | Ackert1 | BMI_M06 | body mass index (BMI) | kg/m<sup>2</sup> | body weight size and growth | 0.29 | 0.01 | < 0.0001 |
| 25036 | Ackert1 | pctfat_M06 | calculated percent fat of body mass | % | body composition | 3.29 | 0.16 | < 0.0001 |
| 25041 | Ackert1 | TTM_M06 | calculated total tissue mass | g | body composition | 5.13 | 0.13 | < 0.0001 |
| 25043 | Ackert1 | TTM_M20 | calculated total tissue mass | g | body composition | 2.18 | 0.24 | < 0.0001 |
| 25046 | Ackert1 | LTM_M06 | calculated weight of lean tissue mass | g | body composition | 2.73 | 0.08 | < 0.0001 |
| 25048 | Ackert1 | LTM_M20 | calculated weight of lean tissue mass | g | body composition | 1.35 | 0.15 | < 0.0001 |
| 25206 | Mills1 | early_apoptotic_M12 | splenocytes that are early apoptotic | % | cell and tissue damage | 2.35 | 0.07 | < 0.0001 |
| 25232 | Mills1 | RBC_micronucl_M12 | RBC with micronuclei | % | cell and tissue damage | 0.1 | 0.01 | < 0.0001 |
| 25236 | Mills1 | all_micronucl_M12 | non-nucleated peripheral blood cells with micronuclei (retic + RBC) | % | cell and tissue damage | 0.11 | 0.01 | < 0.0001 |
| 25602 | Finn1 | 6pct_EtOH | dose of ethanol voluntarily consumed at 6% ethanol in water | g/kg | ingestive preference | 1.04 | 0.12 | < 0.0001 |
| 25605 | Finn1 | 3pct_EtOHsach | dose of ethanol voluntarily consumed at 3% ethanol in water with 0.2% saccharin | g/kg | ingestive preference | 1.81 | 0.12 | < 0.0001 |
| 25606 | Finn1 | 6pct_EtOHsach | dose of ethanol voluntarily consumed at 6% ethanol in water with 0.2% saccharin | g/kg | ingestive preference | 1.6 | 0.17 | < 0.0001 |
| 27011 | Yuan1 | bw_M06 | body weight | g | body weight size and growth | 6.06 | 0.14 | < 0.0001 |
| 27012 | Yuan1 | bw_M12 | body weight | g | body weight size and growth | 1.5 | 0.18 | < 0.0001 |
| 29701 | Korstanje1 | MA_M12 | microalbumin (urine MA) | mg/dL | kidney | 5.2 | 0.56 | < 0.0001 |
| 29711 | Korstanje1 | CREA_M12 | creatinine (urine CREA) | mg/dL | kidney | 13.21 | 1.02 | < 0.0001 |
| 29801 | Zheng1 | Rcompliance | tympanic membrane compliance. right ear | mL | ear | -0.09 | 0.01 | < 0.0001 |
| 29804 | Zheng1 | Rpressure | middle ear pressure at maximum compliance. right ear | daPa | ear | 12.28 | 1.25 | < 0.0001 |
| 30401 | Vinyard1 | max_gape | distance between upper and lower incisor tips at maximum passive jaw opening (maximum gape) | mm | bone | 0.62 | 0.02 | < 0.0001 |
| 30404 | Vinyard1 | condyle_length | length of rostral-caudal condylar articular surface (condyle length) | mm | bone | 0.13 | 0.01 | < 0.0001 |
| 30408 | Vinyard1 | bw | body weight | g | body weight size and growth | 2.1 | 0.07 | < 0.0001 |
| 30480 | Vinyard1 | age | age at test | wks | local experiment parameter | -0.41 | 0.02 | < 0.0001 |
| 32314 | Schonfeld1 | pct_ITT30 | glucose level as percent of baseline. 30 min. insulin tolerance test (insulin i.p.. 5h fast) | % | blood--clinical chemistry | 16.45 | 1.8 | < 0.0001 |
| 32315 | Schonfeld1 | pct_ITT60 | glucose level as percent of baseline. 60 min. insulin tolerance test (insulin i.p.. 5h fast) | % | blood--clinical chemistry | 13.33 | 2.11 | < 0.0001 |
| 32332 | Schonfeld1 | FC | free cholesterol (plasma. 5h fast) | mg/dL | blood--lipids | -3.18 | 0.53 | < 0.0001 |
| 32354 | Schonfeld1 | BW | body weight | g | body weight size and growth | 7.26 | 0.21 | < 0.0001 |
| 32361 | Schonfeld1 | LW | liver weight | g | liver | 0.39 | 0.01 | < 0.0001 |
| 33402 | Shockley1 | BUN | blood urea nitrogen (serum BUN. 5h fast) | mg/dL | blood--clinical chemistry | 2.19 | 0.23 | < 0.0001 |
| 33404 | Shockley1 | GLDH | glutamate dehydrogenase (serum GLDH. 5h fast) | IU/L | blood--clinical chemistry | 9.89 | 1.13 | < 0.0001 |
| 33406 | Shockley1 | CHOL | total cholesterol (serum CHOL. 5h fast) | mg/dL | blood--lipids | 51.35 | 1.41 | < 0.0001 |
| 33407 | Shockley1 | HDL | high density lipoprotein cholesterol (serum HDL. 5h fast) | mg/dL | blood--lipids | 18.47 | 1.38 | < 0.0001 |
| 33437 | Shockley1 | HDL_fat | high density lipoprotein cholesterol (serum HDL. 5h fast) | mg/dL | blood--lipids | 68.54 | 2.04 | < 0.0001 |
| 33440 | Shockley1 | T4_fat | thyroxine (serum T4. 5h fast) | &micro | ungrouped | -2.38 | 0.19 | < 0.0001 |
| 34601 | Rhodes1 | activity_control | distance traveled per day in homecage. without exercise wheel | km/day | behavior | -0.06 | 0 | < 0.0001 |
| 34609 | Rhodes1 | BW_end_control | final body weight. without exercise wheel | g | body weight size and growth | 2.38 | 0.21 | < 0.0001 |
| 35103 | Berndt2 | min_vent_mch10 | minute ventilation (MV) | mL/min/g | respiratory | 6.08 | 0.63 | < 0.0001 |
| 35104 | Berndt2 | min_vent_mch20 | minute ventilation (MV) | mL/min/g | respiratory | 16.37 | 0.77 | < 0.0001 |
| 35115 | Berndt2 | inspiratory_mch5 | inspiratory time (Ti) | s | respiratory | 0.01 | 0 | < 0.0001 |
| 35118 | Berndt2 | inspiratory_slope | inspiratory time (Ti). MCh response index | slope | respiratory | -0.11 | 0.01 | < 0.0001 |
| 35134 | Berndt2 | duty_cycle_saline | duty cycle (Ti/Ttot). saline | ratio | respiratory | -0.02 | 0 | < 0.0001 |
| 35135 | Berndt2 | duty_cycle_mch5 | duty cycle (Ti/Ttot) | ratio | respiratory | -0.01 | 0 | < 0.0001 |
| 35137 | Berndt2 | duty_cycle_mch20 | duty cycle (Ti/Ttot) | ratio | respiratory | 0.02 | 0 | < 0.0001 |
| 35143 | Berndt2 | insp_flow_mch10 | inspiratory flow rate (Vt/Ti) | mL/kg/s | respiratory | 0.15 | 0.02 | < 0.0001 |
| 35144 | Berndt2 | insp_flow_mch20 | inspiratory flow rate (Vt/Ti) | mL/kg/s | respiratory | 0.61 | 0.03 | < 0.0001 |
| 35145 | Berndt2 | insp_flow_slope | inspiratory flow rate (Vt/Ti). MCh response index | slope | respiratory | 0.2 | 0.01 | < 0.0001 |
| 35150 | Berndt2 | exp_flow_mch10 | expiratory flow rate (Vt/Te) | mL/kg/s | respiratory | 0.15 | 0.02 | < 0.0001 |
| 35151 | Berndt2 | exp_flow_mch20 | expiratory flow rate (Vt/Te) | mL/kg/s | respiratory | 0.47 | 0.02 | < 0.0001 |
| 35156 | Berndt2 | resp_flow_mch10 | respiratory flow rate (Vt/Ttot) | mL/kg/s | respiratory | 0.09 | 0.01 | < 0.0001 |
| 35157 | Berndt2 | resp_flow_mch20 | respiratory flow rate (Vt/Ttot) | mL/kg/s | respiratory | 0.27 | 0.01 | < 0.0001 |
| 35162 | Berndt2 | penh_mch5 | enhanced pause (Penh) | respiratory | breathing pattern | 0.35 | 0.02 | < 0.0001 |
| 35163 | Berndt2 | penh_mch10 | enhanced pause (Penh) | respiratory | breathing pattern | 1.04 | 0.06 | < 0.0001 |
| 35170 | Berndt2 | tidal_vol_mch10 | tidal volume (Vt) | mL/kg | respiratory | 0.02 | 0 | < 0.0001 |
| 35171 | Berndt2 | tidal_vol_mch20 | tidal volume (Vt) | mL/kg | respiratory | 0.04 | 0 | < 0.0001 |
| 35184 | Berndt2 | airway_res_mch10 | airway resistance (Rn) (H<sub>2</sub>0 pressure) | cm/mL/s | respiratory | 0.22 | 0.02 | < 0.0001 |
| 35186 | Berndt2 | airway_res_slope | airway resistance (Rn) (H<sub>2</sub>0 pressure). MCh response index | slope | respiratory | 0.07 | 0 | < 0.0001 |
| 35191 | Berndt2 | airway_elas_saline | lung tissue elastance (H) (H<sub>2</sub>0 pressure). saline | cm/mL | respiratory | 5.96 | 0.25 | < 0.0001 |
| 35192 | Berndt2 | airway_elas_mch1 | lung tissue elastance (H) (H<sub>2</sub>0 pressure) | cm/mL | respiratory | 5.19 | 0.35 | < 0.0001 |
| 35196 | Berndt2 | airway_elas_slope | lung tissue dynamic elastance (H) (H<sub>2</sub>0 pressure). MCh response index | slope | respiratory | -0.04 | 0 | < 0.0001 |
| 35703 | Lightfoot1 | activity_speed | wheel running activity. mean speed | m/min | behavior | 2.41 | 0.25 | < 0.0001 |
| 35704 | Lightfoot1 | bw | body weight at the start of testing | g | body weight size and growth | 3.88 | 0.1 | < 0.0001 |
| 35780 | Lightfoot1 | age | age at testing | d | local experiment parameter | -4.37 | 0.24 | < 0.0001 |
| 36801 | Vulpe1 | copper | copper (Cu) content in liver (dry weight) | &micro | ungrouped | -2.81 | 0.14 | < 0.0001 |
| 36803 | Vulpe1 | zinc | zinc (Zn) content in liver (dry weight) | &micro | ungrouped | -8.26 | 0.62 | < 0.0001 |
